# Near‐Infrared Light‐Induced Reversible Deactivation Radical Polymerization: Expanding Frontiers in Photopolymerization

**DOI:** 10.1002/advs.202304942

**Published:** 2023-09-26

**Authors:** Zilong Wu, Cyrille Boyer

**Affiliations:** ^1^ Cluster for Advanced Macromolecular Design and Australian Centre for NanoMedicine School of Chemical Engineering The University of New South Wales Sydney NSW 2052 Australia

**Keywords:** controlled/living radical polymerization, near‐infrared light, photochemistry, photopolymerization, reversible deactivation radical polymerization, upconversion nanoparticles

## Abstract

Photoinduced reversible deactivation radical polymerization (photo‐RDRP) or photoinduced controlled/living radical polymerization has emerged as a versatile and powerful technique for preparing functional and advanced polymer materials under mild conditions by harnessing light energy. While UV and visible light (*λ* = 400–700 nm) are extensively employed in photo‐RDRP, the utilization of near‐infrared (NIR) wavelengths (*λ* = 700–2500 nm) beyond the visible region remains relatively unexplored. NIR light possesses unique properties, including enhanced light penetration, reduced light scattering, and low biomolecule absorption, thereby providing opportunities for applying photo‐RDRP in the fields of manufacturing and medicine. This comprehensive review categorizes all known NIR light‐induced RDRP (NIR‐RDRP) systems into four mechanism‐based types: mediation by upconversion nanoparticles, mediation by photocatalysts, photothermal conversion, and two‐photon absorption. The distinct photoinitiation pathways associated with each mechanism are discussed. Furthermore, this review highlights the diverse applications of NIR‐RDRP reported to date, including 3D printing, polymer brush fabrication, drug delivery, nanoparticle synthesis, and hydrogel formation. By presenting these applications, the review underscores the exceptional capabilities of NIR‐RDRP and offers guidance for developing high‐performance and versatile photopolymerization systems. Exploiting the unique properties of NIR light unlocks new opportunities for synthesizing functional and advanced polymer materials.

## Graphical abstract

Near‐infrared (NIR) light has unique properties, including enhanced light penetration, reduced light scattering, and low absorption by biomolecules, thereby providing opportunities for applying reversible deactivation radical polymerization (RDRP) in manufacturing and medicine. This review summarizes the mechanisms and applications of various NIR light‐induced RDRP systems.

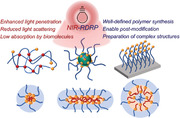

## 1 Introduction

Since Ciamician's visionary address over a century ago, photochemistry has continuously evolved, fueling innovation across various research fields.^[^ ^1^ ^]^ Alongside the strong emphasis on using sunlight as a renewable energy source,^[^ ^2^ ^]^ light‐mediated chemical syntheses have also garnered considerable attention through the years.^[^ ^3^ ^]^ Researchers are increasingly focusing on developing photochemical systems as alternatives to traditional thermal methods. Unlike reactions initiated by heat, photochemical systems employ light energy for chemical synthesis and can operate at room temperature.^[^ ^4^ ^]^ Moreover, the energy from photons can be harvested by light‐absorbing photosensitizers or photocatalysts (PCs) enabling efficient and selective chemical transformations.^[^ ^5^ ^]^

Polymeric materials have become ubiquitous in our daily lives, finding applications in a wide range of fields in paint, packaging, and construction materials, as well as advanced polymers employed in microelectronics and medicine. While these established products and their synthetic methods have been refined and optimized for their specific applications, the majority of commercial polymers are produced via conventional chain growth (conventional radical polymerization) or step growth polymerization. These conventional methods have their limitations to impart precise control over synthesized macromolecular structures. For example, in conventional chain growth polymerization, the occurrence of irreversible chain transfer and termination reactions is unavoidable, leading to broad molecular distributions of polymers and “dead” polymer chains. Therefore, it is challenging to introduce functional blocks within polymer chains via conventional polymerization, further limiting the properties of polymeric materials. To overcome these issues, polymerization systems regulated by various control agents have been developed in recent decades for the reversible deactivation of propagating radicals to “dormant” species, efficiently minimizing radical termination and imparting most of the “living” characteristics in radical polymerization.^[^ ^6^ ^]^ While these systems were commonly referred to as controlled/living radical polymerization (CLRP), IUPAC has recommended the use of the terminology: reversible‐deactivation radical polymerization (RDRP). RDRP reflects the presence of some irreversible chain termination reactions, despite the “living” characteristics exhibited by these systems. Notably, the RDRP technique enables the facile synthesis of polymers with well‐defined and complex architectures, holding great potential for broad applications.^[^ ^6^, ^7^ ^]^

Echoing developments in photochemistry, the RDRP process has been demonstrated to be regulated by light energy. A wide range of photoinduced reversible deactivation radical polymerization (photo‐RDRP) systems regulated by wavelengths in the UV (*λ* = 100–400 nm) and visible (*λ* = 400–700 nm) range have been reported, creating various exciting applications. First, photo‐RDRP systems enable the preparation of functional and complex materials under light irradiation at room temperature. The independence of photoinitiation from reaction temperatures enables polymer preparation at mild conditions, facilitating a diverse range of applications, even under biological conditions.^[^ ^8^ ^]^ Moreover, owing to the retention of functional groups in polymer material, photo‐RDRP can be utilized to reactivate the polymer end groups under light irradiation, enabling postmodification with spatiotemporal control.^[^ ^9^ ^]^ While the use of visible light offers several advantages over high‐energy UV light, such as a safer operating environment and fewer unwanted side reactions,^[^ ^10^ ^]^ visible light presents low penetration depth through nontransparent barriers,^[^ ^11^ ^]^ limiting its applicability in some fields.

**Figure 1:**
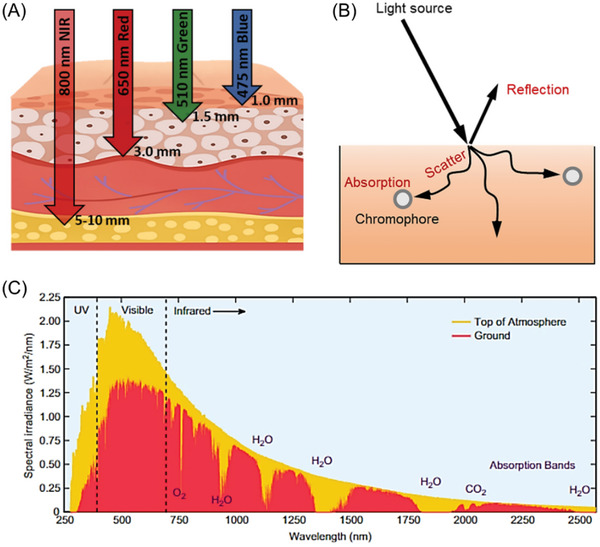
A) The enhanced penetration depth of near‐infrared (NIR) through soft‐tissue barriers in comparison with UV and visible light. Reproduced with permission.^[^ ^15^ ^]^ Copyright 2017, John Wiley & Sons. B) Light reflection, scattering, and absorption by molecules within the tissue (chromophores). C) Solar spectra consisting of UV, visible, and near‐infrared (NIR) light. Reproduced with permission.^[^ ^16^ ^]^ Copyright 2013, Elsevier. Data from United States Department of Energy, National Renewable Energy Laboratory, Reference Solar Spectral Irradiance: ASTM G‐173.

Near‐infrared (NIR) light offers some remarkable advantages in comparison to visible or UV light. One significant advantage is its ability to penetrate through opaque barriers, as illustrated in **Figure** **1**A. Additionally, NIR light exhibits reduced Rayleigh scattering as it is proportional to 1/*λ* ^4^ (*λ* is the wavelength of incident light), meaning that shorter wavelengths (such as UV and visible light) are more prone to scattering. This characteristic of NIR light promotes a more even distribution of light intensity within the reaction media (Figure 1B). As a result, it enhances the efficiency and precision of photopolymerization and photochemical reactions in heterogeneous systems. Moreover, the low absorption of NIR light by the most commonly used solvents, biomolecules, and chromophores (Figure 1B) makes it highly suitable for bioapplications. In this regard, expanding the toolbox of NIR light‐induced polymerization can open new opportunities for the preparation of advanced materials and the functionalization of biomolecules.^[^ ^12^ ^]^ Furthermore, considering that more than 50% of the solar spectrum consists of longer wavelengths beyond the visible spectrum (Figure 1C),^[^ ^13^ ^]^ harnessing the photochemical capabilities of NIR light allows for more optimized utilization of sunlight.^[^ ^14^ ^]^ Expanding the range of photopolymerization reactions to NIR wavelengths provides a pathway for leveraging the abundant and readily available solar energy, offering potential advancements in sustainable and efficient polymerization methodologies.

NIR light‐induced RDRP (NIR‐RDRP) has emerged as a promising approach, enabling the synthesis of well‐defined polymers, nanoparticles, and polymeric networks through opaque systems,^[^ ^17^ ^]^ by harnessing its enhanced penetration through materials. This capability has been utilized in advanced fabrication techniques like 3D two‐photon laser printing (2PLP), enabling the precise fabrication of objects with high resolution and “living” properties that can be further postmodified through polymer chain extension.^[^ ^18^ ^]^ Additionally, the reduced scattering of longer wavelengths (NIR light) minimizes the gradient of light intensity in colloidal media, facilitating the development and scaling of heterogeneous photopolymerization systems for the production of functional polymers with complex structures.^[^ ^17c^ ^]^ The application of NIR‐RDRP is particularly promising in the preparation of bioactive polymers, such as protein–polymer conjugates for medical treatments, because the low absorption of biomolecules by NIR light enhances the feasibility of incorporating bioactive components into polymers.^[^ ^19^ ^]^ These contributions pave the way for the future applications of RDRP for the preparation of advanced polymer materials, especially in some fields requiring high light penetration.

The review aims to provide a comprehensive overview of established NIR‐RDRP systems, highlighting their advantages and limitations. In the first part, we discuss the photoinitiation pathways of NIR‐RDRP by cataloging into four different systems: upconversion nanoparticle (UCNP)‐mediated systems, photocatalyst (PC)‐mediated systems, polymerization via photothermal conversion, and polymerization via two‐photon absorption. Notably, various NIR‐RDRP systems necessitate specific light sources. For instance, UCNP‐mediated systems typically require 980 nm continuous wave (CW) lasers for photoactivation, whereas PC‐mediated systems make use of 700–800 nm light‐emitting diodes (LEDs). It is worth noting that light emitted from 980 nm CW lasers offers superior penetration and more precise spatial control compared to 700–800 nm LEDs. However, the latter devices present the following advantages, such as lower light intensity, safer operating conditions, larger irradiation areas, and lower costs. Consequently, the choice of light source can be tailored to meet the practical requirements of NIR‐RDRP applications. Subsequently, we summarize the diverse potential applications of NIR‐RDRP in various areas. These include the preparation of advanced materials, including nanoparticles and polymer brushes,^[^ ^17^ ^]^ 3D printing for precise control of the polymeric network,^[^ ^18^, ^20^ ^]^ drug delivery systems,^[^ ^21^ ^]^ and membrane reactors.^[^ ^19^ ^]^ Finally, we provide a brief discussion on the current challenges and future perspectives in the development and application of NIR‐RDRP, outlining the potential directions for further research and advancements in the field.

## 2 Mechanisms of NIR‐RDRP

**Figure 2:**
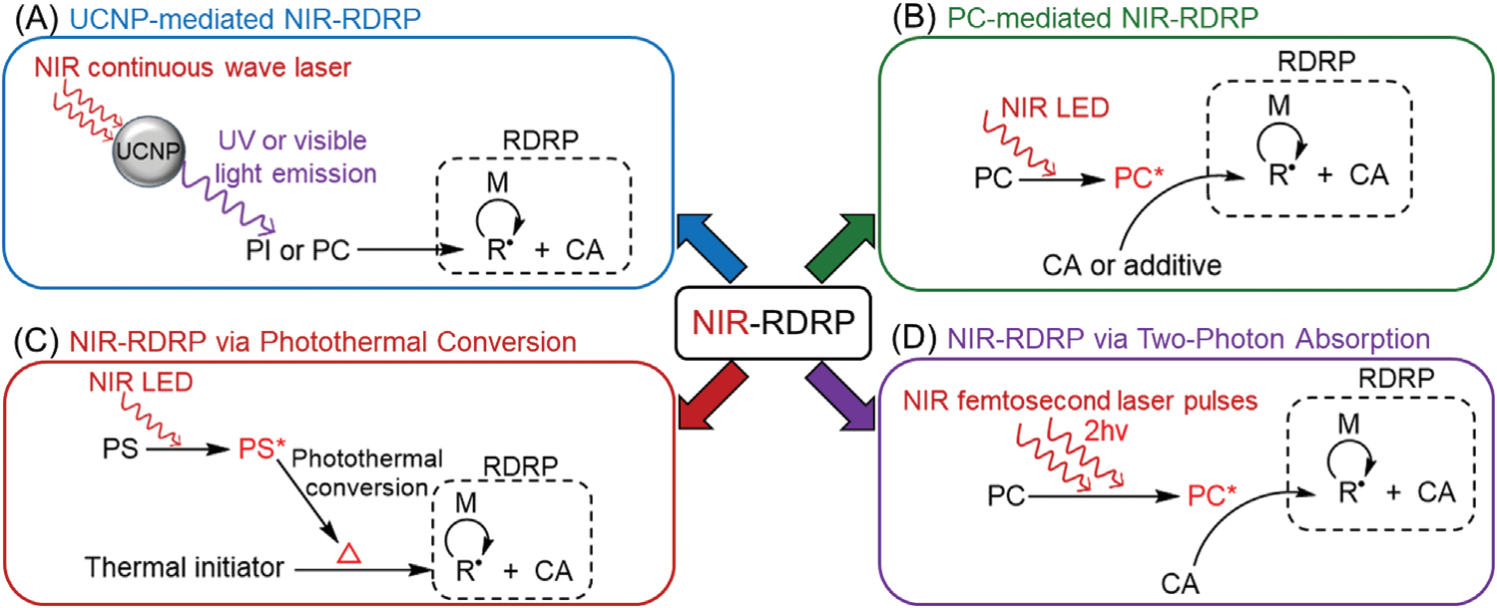
Various photoinitiation mechanisms of NIR‐RDRP. Note: near‐infrared (NIR), upconversion nanoparticle (UCNP), photoinitiator (PI), photocatalyst (PC), reversible‐deactivation radical polymerization (RDRP), RDRP control agent (CA), light‐emitting diode (LED), and photosensitizer (PS).

The field of photochemistry has witnessed significant development in utilizing inorganic nanoparticles^[^ ^22^ ^]^ and conjugated organic molecules^[^ ^23^ ^]^ for harnessing NIR wavelengths in chemical reactions. Recently, these NIR‐absorbing compounds have been integrated into photo‐RDRP systems, allowing for the synthesis of well‐defined polymers under NIR irradiation. In this review, we identify four distinct photoinitiation pathways of RDRP under NIR irradiation (**Figure** **2**).

1. UCNP‐mediated systems. These systems utilize UCNPs to convert low‐energy NIR light into higher‐energy photons, enabling the initiation or activation of RDRP reactions.^[^ ^17^, ^21^, ^24^ ^]^
2. PC‐mediated systems. PC‐based systems leverage the photocatalytic properties of specific materials to initiate RDRP under NIR light.^[^ ^22^, ^23^, ^25^ ^]^
3. Polymerization via photothermal conversion. This approach involves the use of compounds that can efficiently convert NIR light into heat, leading to localized temperature increase and subsequent initiation of RDRP by the decomposition of initiators.^[^ ^26^ ^]^
4. Polymerization via two‐photon absorption. NIR light‐induced polymerization can also be achieved through two‐photon absorption, where the simultaneous absorption of two low energy photons initiates RDRP reactions.^[^ ^18^, ^27^ ^]^

When exposed to NIR CW laser, lanthanide‐doped UCNPs can emit UV or visible light by multiple‐photons conversions.^[^ ^22a^ ^]^ Perhaps, one of the first examples of photopolymerization was demonstrated for the preparation of dental resin‐containing filler.^[^ ^28^ ^]^ Jogo and co‐workers exploited the rare earth‐doped (Y_2_O_3_) particles, which acted as fillers and illuminators to cure the dental resin by emitting blue light under the irradiation of NIR light. While this first example was using a conventional radical polymerization, more recently, the use of these UCNPs to activate RDRP was investigated. In UCNP‐mediated NIR‐RDRP systems (Figure 2A), the emitted UV or visible light can be then absorbed by a photoinitiator (PI) or PC, initiating RDRP in the presence of control agents (CAs). In the second mechanism (Figure 2B), a photoinduced electron/energy transfer (PET) process takes place between the PC and either a CA or an additive after being excited by NIR wavelengths. This PET process generates initiating radicals under light, which then activate the RDRP reaction.^[^ ^29^ ^]^ In the third process (Figure 2C), a photosensitizer is used as a photothermal conversion agent, converting NIR irradiation into heat. Thermal energy activates the decomposition of thermal initiators, resulting in the formation of initiating species. In the final mechanism (Figure 2D), under the irradiation of NIR femtosecond laser pulses, specific PCs or photoinitiators can be photoactivated via a two‐photon absorption (TPA) process,^[^ ^30^ ^]^ activating RDRP.

Importantly, specific NIR light sources are required for photoinitiation in some of these mechanisms. For example, the irradiation of CW NIR lasers is necessary for UCNPs to convert these low‐energy photons to UV or visible light through multiphonon‐assisted relaxations (Figure 2A).^[^ ^24^, ^31^ ^]^ By contrast, PC‐mediated processes (Figure 2B) and photothermal conversion mechanisms (Figure 2C) are one‐photon processes and do not rely on specific light sources.^[^ ^8^, ^25^, ^32^ ^]^ In these cases, NIR LEDs are commonly used because LEDs offer several advantages, such as lower light intensity, larger exposure areas, safer operation conditions, and cheaper prices. In the TPA process, a femtosecond laser pulse is required due to the short‐lived intermediated state (typically 10^−15^ s) in TPA.^[^ ^30^ ^]^ This type of laser pulse is more expensive compared to CW NIR lasers (Figure 2D).

**Figure 3:**
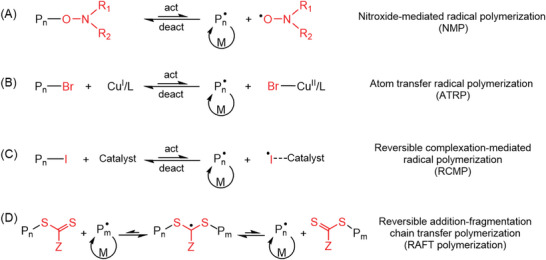
Various reversible deactivation processes of initiating radical in NIR‐RDRP, including ATRP, RCMP, and RAFT processes. Control agents (P_n_–X) with various X groups (red‐colored part) used in RDRP.

In addition to the photoinitiation pathways, NIR‐RDRP can also be classified based on the different reversible deactivation processes of propagating radicals during polymerization (**Figure** **3**). Four main processes are commonly employed, including nitroxide‐mediated polymerization (NMP),^[^ ^18^, ^27^ ^]^ atom transfer radical polymerization (ATRP),^[^ ^17^, ^24^, ^25^ ^]^ reversible complexation mediated polymerization (RCMP),^[^ ^25^, ^32^ ^]^ and reversible addition–fragmentation chain‐transfer (RAFT) polymerization.^[^ ^8^, ^19^, ^20^, ^22^, ^23^, ^25^, ^32^, ^33^ ^]^ In NMP systems (Figure 3A), the homolysis of the C─O bond in the functional alkoxyamine group occurs under high temperature or light irradiation, resulting in the generation of an initiating radical and a nitroxide radical.^[^ ^34^ ^]^ The initiating radical then drives chain propagation, while propagating radicals can be deactivated by nitroxide radicals to regenerate alkoxyamine groups. ATRP (Figure 3B) involves an equilibrium between propagating radicals (alkyl macromolecular radical, P_n_^•^) and dormant species (alkyl halides/macromolecular species, P_n_–Br).^[^ ^35^ ^]^ Under specific reaction conditions, the catalyst, often based on copper(I) or another transition metal complex in its lower oxidation state, is employed to periodically activate the dormant species to generate initiating radicals. These radicals can react with monomers or be reversibly terminated by a reaction with the catalyst in a higher oxidation state, such as copper(II).

In RCMP, organic amines are commonly used as catalysts to facilitate the abstraction of iodine from an iodine compound (P_n_–I). This abstraction process leads to the reversible generation of P_n_^•^ and a complex comprising an iodine radical and the catalyst (^•^I–catalyst) (Figure 3C). In RAFT polymerization (Figure 3D), the highly reactive C═S bonds can react with propagating radicals (P_n_^•^) to form intermediate species (P_m_–S–(C^•^–Z)–S–P_n_), which subsequently undergo fragmentation to yield P_m_^•^. In contrast to NMP, ATRP, and RCMP, the chain transfer process, i.e., rapid transfer of radicals from one chain to another chain, occurs in RAFT polymerization.^[^ ^36^ ^]^

### 2.1 UCNP‐Mediated NIR‐RDRP

Lanthanide ions possess unique magnetic and optical properties attributed to their partially filled 4f orbitals (4f^n^5s^2^5p^6^ (*n* = 0–14)). Although 4f electrons of lanthanide ions are shielded by the outer 5s and 5p shells, the arrangement of the host lattice can influence the transition of these inner electrons, potentially breaking the parity‐forbidden rule and allowing successful 4f electron transitions.^[^ ^37^ ^]^ These transitions play a key role in lanthanide‐based upconversion luminescence (UCL) processes.^[^ ^38^ ^]^ UCL processes can be identified by mechanisms such as excited state absorption, energy transfer upconversion (ETU), photon avalanche, cooperative energy transfer, and energy migration‐mediated upconversion.^[^ ^22^, ^39^ ^]^ ETU, which exhibits high upconversion efficiency,^[^ ^40^ ^]^ is commonly employed in the design of UCNPs.

**Figure 4:**
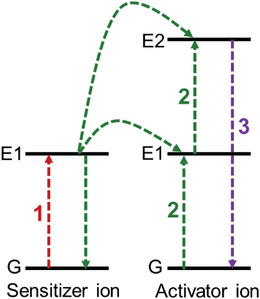
Energy transfer upconversion (ETU) process for UCNPs.

In ETU, a sensitizer ion is excited from its ground state to a metastable level E1 by absorbing a photon (**Figure** **4**, Step 1). E1 of the sensitizer then transfers its energy to the ground state (G) and excited state (E1) of an activator ion, exciting the activator to its upper emitting state E2 (Figure 4, Step 2). Meanwhile, the sensitizer ion relaxes back to the ground state (G) twice (Figure 4, green down arrow). As a result, the emission of a high‐energy photon occurs in Step 3.

Yb^3+^ has a sufficiently large absorption in the NIR region at ≈975 nm, which is normally used as the sensitizer in combination with Tm^3+^ or Er^3+^ activator in UCNPs.^[^ ^41^ ^]^ Notably, the optimized concentration of Yb^3+^ ions can remain high, for example, 20–100% Yb^3+^ in fluoride nanoparticles, without causing detrimental cross‐relaxations owing to its two energy level structure. In addition, the upconversion efficiency of ETU depends on the average distance between neighboring sensitizer‐activator pairs, which is determined by dopant concentrations. Besides sensitizer/activator ion pairs, the selection of appropriate host materials is essential for efficient upconversion emissions. The primary loss pathway for upconversion emissions is the phonon‐induced nonradiative process, which occurs through multiphonon‐assisted relaxations.^[^ ^31^ ^]^ During this process, the energy difference between the higher and lower energy levels is converted into numerous lattice phonons. To date, various host materials have been investigated in the construction of UCNPs, including nanophosphors encompass oxides (e.g., Y_2_O_3_ and ZrO_2_),^[^ ^42^ ^]^ fluorides (e.g., NaYF_4_),^[^ ^21^, ^24^ ^]^ oxysulfide (e.g., Y_2_O_2_S),^[^ ^43^ ^]^ and oxychlorides (e.g., GdOCl).^[^ ^44^ ^]^ Among them, fluoride host materials exhibit the highest upconversion efficiency owing to the minimized nonradiative losses. Therefore, Yb^3+^/Tm^3+^ and Yb^3+^/Er^3+^ (sensitizer/activator) are commonly used in combination with fluoride host materials (e.g., NaYF_4_). For example, NaYF_4_:Yb^3+^, Tm^3+^ (host material: sensitizer ion, activator ion) and NaYF_4_:Yb^3+^, Er^3+^ have been intensively developed in UCNPs, owing to their efficient upconversion of host materials and high optimized concentrations of activators.

Leveraging the enhanced light penetration of NIR irradiation, UCNPs can be excited through nontransparent barriers to induce chemical reactions. Furthermore, UCNPs emit luminescence inside the sample, requiring only a short optical transmission before absorption occurs.^[^ ^24c^ ^]^ These advantages have enabled the use of UCNPs for assisting radical polymerization and photo‐RDRP under NIR light irradiation (**Table** **1**).^[^ ^17^, ^21^, ^24^ ^]^

**Table 1:** Summary of UCNP‐mediated photo‐RDRP systems.

# and refs.	UCNP	Mechanism	Light source and intensity (*I*)	Polymerization performance: conversion (*α*) and dispersity (*Đ*)	Monomer versatility	Solvents
1^[^ ^24a^ ^]^	NaYF_4_:Tm/Yb@NaYF_4_	ATRP (Figure 5A)	24 W 974 nm NIR CW laser	Methyl methacrylate (MMA): *α* = 37% in 2 h *Đ* = 1.8	Acrylate and methacrylate	Toluene
2^[^ ^24b^ ^]^	NaYF_4_:30%Yb/1%Tm	ATRP (Figure 5B)	980 nm NIR CW laser (*I* = 4 W cm^−2^)	Methyl acrylate: *α* = 58% in 24 h *Đ* = 1.19	Acrylate, acrylonitrile, and methacrylate	DMSO, acetonitrile, DMF, and water
3^[^ ^17b^ ^]^	NaYF_4_:Yb/Tm@SiO_2_@N‐CDs	ATRP (Figure 5C)	980 nm NIR CW laser (1.5 W cm^−2^)	2‐Hydroxyethyl acrylate: *α* ≈ 70% in 5 h *Đ* ≈ 1.25	Acrylate and methacrylate	Acetonitrile, DMF, DMAc, and water
4^[^ ^24c^ ^]^	NaYF_4_:Yb/Tm	RAFT (Figure 6A)	2 W 980 nm CW NIR laser	Butyl acrylate: *α* = 87% in 7 h *Đ* = 1.20	Acrylate, methacrylate, and vinyl acetate	Cyclohexane
5^[^ ^21^ ^]^	NaYF_4_:25%Yb/0.5%Tm@NaYbF_4_:50%Gd@NaNdF_4_:10%Yb@NaYF_4_	RAFT (Figure 6A)	808 nm NIR CW laser (12 W cm^−2^)	N/A	Acrylate and methacrylate	DMF
6^[^ ^24d^ ^]^	NaYF_4_:Yb/Tm	RAFT (Figure 6A)	980 nm NIR CW laser (1.5 W cm^−2^)	MMA: *α* ≈ 80% in 20 h *Đ* ≈ 1.20	Acrylate and methacrylate	DMSO
7^[^ ^24e^ ^]^	NaYF_4_:18%Yb/0.5%Tm@SiO_2_‐OEC	RAFT (Figure 6B)	980 nm NIR CW laser (16.8 W cm^−2^)	MMA: *α* = 23.6% in 2 h *Đ* = 1.15	Methacrylate	DMF
8^[^ ^24g^ ^]^	NaYF_4_:Yb/Er/Tm/Gd	RAFT (Figure 7)	980 nm NIR CW laser (9 W cm^−2^)	MMA: *α* = 85.5% in 24 h *Đ* = 1.24	Acrylate, acrylamide and methacrylate	DMSO, DMF

Note: CW: continuous wave laser.

#### 2.1.1 UCNP‐Mediated NIR‐ATRP

**Figure 5:**
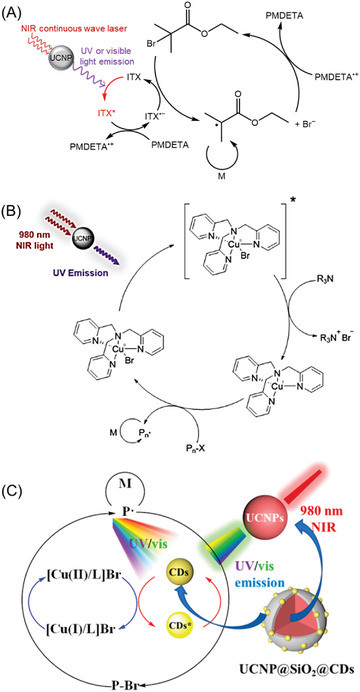
A) Proposed mechanism of UCNP‐mediated metal‐free photo‐ATRP under NIR light irradiation. B) Proposed mechanism for photo‐ATRP under NIR irradiation assisted by UCNPs. Reproduced with permission.^[^ ^24b^ ^]^ Copyright 2020, American Chemical Society. C) Proposed mechanism for photo‐ATRP under UV/vis and NIR irradiation assisted by UCNP@SiO_2_@NCDs. Reproduced with permission.^[^ ^17b^ ^]^ Copyright 2022, American Chemical Society.

In 2017, Strehmel and co‐workers reported the first NIR light‐induced ATRP (NIR‐ATRP) example utilizing NaYF_4_:Tm/Yb@NaYF_4_ UCNPs to activate polymerization of methyl methacrylate (MMA) in toluene under the irradiation of 974 nm laser (Table 1, #1).^[^ ^24a^ ^]^ In the proposed mechanism, isopropyl thioxanthone (ITX) is excited by UV light emitted from UCNPs to gain an electron from *N,N,N′,N″,N″*‐pentamethyldiethylenetriamine (PMDETA), yielding ITX^•−^ (**Figure** **5**A). Subsequently, electron transfer from ITX^•−^ to α‐bromo(iso‐butyl) ethylester (α‐BrBuEt) results in the generation of carbon initiating radicals, activating metal‐free photo‐ATRP. During chain propagation, PMDETA^•+^ and Br^−^ effectively deactivate propagating radicals, thereby regenerating PMDETA and P_n_–Br. Although control of polymerization became better than free radical polymerization as indicated by a lower dispersity (*Đ*) value (*Đ*_ATRP_ = 1.8 vs *Đ*_FRP_ = 6.2), this system still suffers from slow polymerization rates and poor livingness (*Đ* = 1.8).

To improve the controlled characteristics of synthesized polymers, Pan's group introduced the use of β‐NaYF_4_:30%Yb^3+^, 1%Tm^3+^ UCNPs for photo‐ATRP under NIR light. The “living” character of synthesized polymers was demonstrated by achieving low *Đ* values (<1.2). Moreover, this NIR‐ATRP system showed versatility in different solvents (Table 1, #2), including DMSO, acetonitrile (MeCN), DMF, and water. In the photoinitiation mechanism, UCNPs are excited by a 980 nm laser, resulting in the emission of UV light, which activates the ATRP process.^[^ ^24b^ ^]^ Excited by UV light, Cu(II)/(TPMA)Br catalyst is reduced by the excess ligand, generating Cu(I)/(TPMA) (Figure 5B). Subsequently, Cu(I)/(TPMA) activates P_n_–Br to generate the initiating species P_n_^•^, leading to chain propagation. Meanwhile, the excess Cu(II)/(TPMA)Br plays the role of deactivator by reacting with propagating radicals P_n_^•^ to regenerate Cu(I)/(TPMA) and P_n_–Br, completing the catalytic cycle. Various monomers from different families, including methyl acrylate (MA) ethyl acrylate (EA), *tert*‐butyl acrylate (tBA), MMA, acrylonitrile (AN), oligo(ethylene glycol) methyl ether methacrylate (OEGMA_500_, *M*_n_ = 500), and oligo(ethylene glycol) methyl ether acrylate (OEGA_480_, *M*_n_ = 480), were successfully polymerized. To demonstrate the enhanced penetration of NIR light, photopolymerization was conducted through a 1.2 mm thickness pig skin, achieving 88% monomer conversion of MA with excellent control (*Đ* = 1.16) after 36 h. By contrast, the pig skin prevented ATRP under UV light due to the limited penetration of these high‐energy photons.

In a subsequent study, the Pang group introduced a novel heterogeneous photocatalyst (UCNP@SiO_2_@N‐CDs) by combining pyridine nitrogen‐doped carbon dots (N‐CDs) and UCNPs for NIR‐ATRP (Table 1, #3).^[^ ^17b^ ^]^ The notable improvement of UCNP@SiO_2_@N‐CDs compared to previous UCNPs in photo‐ATRP is its ability to absorb a broad range of wavelengths, ranging from UV to NIR light. Experimental results demonstrated the successful activation of photo‐ATRP under different irradiation wavelengths, including blue, green, red, and NIR light, enabling the synthesis of well‐defined polymers with a low polydispersity index (*Đ* < 1.3). The proposed mechanism indicates that the CDs work as PCs and are excited by UV and visible light, either from external light irradiation or light emission from UCNPs. As a result, the excited CDs interact with [Cu(II)/L]Br triggering a PET process, resulting in the generation of Cu(I)/L species and activation of the ATRP process (Figure 5C).

#### 2.1.2 UCNP‐Mediated‐RAFT Polymerization

**Figure 6:**
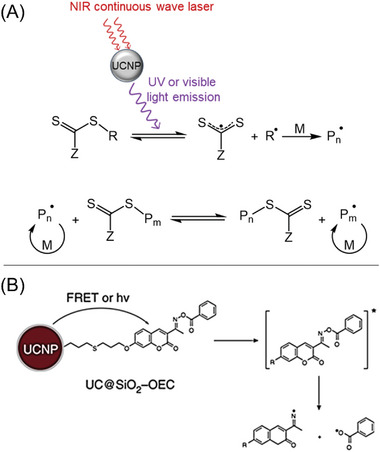
A) Proposed mechanism of UCNP‐mediated photoiniferter RAFT polymerization under NIR CW laser irradiation. B) The mechanism of photopolymerization by using UC@SiO_2_‐OEC. Adapted with permission.^[^ ^24e^ ^]^ Copyright 2019, Springer Nature.

In 2016, Zhu and co‐workers were the first to employ UCNPs as photosensitizers for the activation of RAFT polymerization under NIR irradiation (Table 1, #4).^[^ ^24c^ ^]^ They utilized NaYF_4_:Yb^3+^, Tm^3+^ UCNPs, which emit light in the range of 325–380 and 425–500 nm when irradiated with an NIR CW laser. This light‐emitting spectrum of NaYF_4_:Yb^3+^, Tm^3+^ UCNP matches the absorption of trithiocarbonates and xanthates, enabling a direct photoiniferter RAFT polymerization process (**Figure** **6**A). Under UV and blue light irradiation, the RAFT agents undergo homolytic cleavage (or photolysis),^[^ ^45^ ^]^ followed by chain propagation and chain transfer. This NIR light‐induced RAFT (NIR‐RAFT) system exhibited great versatility toward various monomers, including *n*‐BA, MMA, and vinyl acetate, yielding polymers with narrow MWDs. Furthermore, high end‐group fidelity was verified by ^1^H NMR spectroscopy and the matrix‐assisted laser desorption ionization time‐of‐flight mass spectrometry. Subsequently, independent research groups led by Luan and Pang also reported similar approaches for surface‐initiated polymerization using the photoiniferter RAFT mechanism (Figure 6A) assisted by UCNPs in 2017, and 2021, respectively (Table 1, #5 and 6).^[^ ^21^, ^24^ ^]^ In these contributions, functional polymer shell nanohybrids were successfully prepared under NIR irradiation, which will be introduced in Section 3.3.

In order to improve the efficiency of photoinitiation in polymerization, Yagci's group introduced a novel approach by incorporating an oxime‐ester coumarin (OEC) group onto the surface of UCNPs (NaYF_4_:18%Yb^3+^, 0.5%Tm^3+^ UCNPs, Table 1, #7).^[^ ^24e^ ^]^ This innovative design resulted in the formation of a dandelion‐like photoinitiator. Under NIR light irradiation, UCNPs emitted UV, and visible light, which were absorbed by OEC, leading to the cleavage of oxime ester groups and the generation of initiating species in solution (Figure 6B). The incorporation of PI onto UCNPs enhanced the quantum yield of photodissociation of the photoinitiator. This process was successfully employed to activate a thiol–ene reaction and RAFT polymerization of MMA in DMF, enabling the preparation of PMMA with a low dispersity (*Đ*  =  1.15).

**Figure 7:**
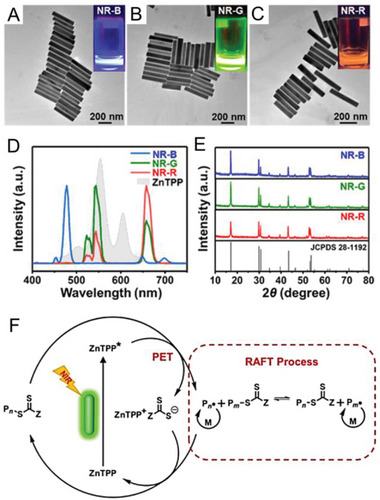
TEM images of UCNPs A) NaYF_4_:30%Yb^3+^, 1%Tm^3+^, 30% Gd^3+^ NR (NR‐B), B) NaYF_4_:18%Yb^3+^, 2%Er^3+^, 60%Gd^3+^ (NR‐G), and C) NaYF4:2%Yb^3+^, 2%Er^3+^, 2%Tm^3+^, 30%Gd^3+^ (NR‐R). The insets show the emission of UNCs under 980 nm laser irradiation. D) Fluorescence spectra of UCNPs dissolved in DMSO with the same concentration under 980 nm laser irradiation (the gray area is the absorption spectrum of ZnTPP). E) XRD patterns of NR‐B, NR‐G, and NR‐R. F) Proposed mechanism for NIR PET‐RAFT polymerization in the presence of UCNPs and the RAFT agents. Reproduced with permission.^[^ ^24g^ ^]^ Copyright 2022, American Chemical Society.

The properties of NaYF_4_:Yb^3+^, Tm^3+^, Er^3+^, and Gd^3+^ UCNPs can be easily modified by changing synthesis conditions and doping gadolinium (Gd^3+^) concentration. Exploiting this advantage, Pang's group investigated a library of UCNPs with various morphologies and luminescence emission to mediate photoinduced electron/energy transfer reversible addition–fragmentation chain transfer (PET‐RAFT) polymerization.^[^ ^24g^ ^]^ By increasing reaction temperature, the morphology transformation of NaYF_4_:Tm^3+^ was manipulated from α cubic nanoparticle (α‐NP) to β hexagonal nanoparticle (β‐NP). Moreover, β‐NP morphology evolved to β‐hexagonal nanorod (β‐NR) by the addition of gadolinium (Gd^3+^) in UCNPs. Interestingly, the highest to lowest luminescent intensity of UCNPs was determined in the following order of β‐NR > β‐NP > α‐NP, which was attributed to fewer surface defects of 0D UCNPs in comparison to 1D structures.^[^ ^46^ ^]^ Zinc tetraphenyl porphin (ZnTPP) was used as PC for PET‐RAFT polymerization (Table 1, #8).^[^ ^47^ ^]^ By varying the doping elements, various colors of light emission of UCNPs, including blue (NR‐B), green (NR‐G), and red (NR‐R), were synthesized (**Figure** **7**A–C) with a high crystallinity as confirmed by X‐ray diffraction (XRD) analysis (Figure 7E). Specifically, among them, NR‐G demonstrated the most efficient activation for ZnTPP‐mediated PET‐RAFT polymerization. This enhanced efficiency was attributed to the high quantum yield of green light emission^[^ ^48^ ^]^ as well as the maximum overlap between the emission spectrum of UCNPs and the absorption spectrum of ZnTPP (Figure 7D). In the proposed mechanism (Figure 7F), ZnTPP is excited by the visible light emitted from UCNPs under NIR irradiation. Subsequently, a photoinduced electron transfer process occurs from excited ZnTPP to RAFT agent, generating a PC cationic radical (PC^•+^) and an initiating radical/residual RAFT anionic radical binary pair (P_n_^•^/RAFT agent^•−^). Subsequently, chain propagation occurs in the presence of P_n_^•^, and propagating radicals are deactivated by RAFT agents simultaneously. In the back electron transfer reaction from P_n_^•^/RAFT agent^•−^ to PC^•+^, the P_n_–RAFT agent and PC are regenerated. In addition to controlling a broad range of monomers, including (meth)acrylates, and (meth)acrylamides, this system has the advantage of not requiring deoxygenation. Unlike some polymerization processes that are sensitive to the presence of oxygen, the PET‐RAFT polymerization system using ZnTPP and UCNPs does not require special equipment or procedures for deoxygenation.

### 2.2 PC‐Mediated NIR‐RDRP

**Figure 8:**
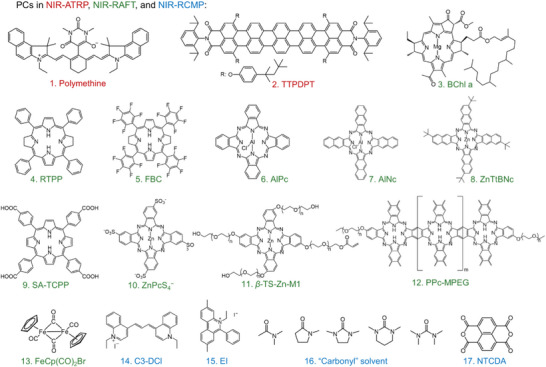
Various PCs used in NIR‐RDRP, including NIR‐RAFT polymerization, NIR‐ATRP, and NIR‐RCMP.

Recently, there has been a growing interest in utilizing NIR light‐excitable chromophores for photo‐RDRP systems. These chromophores are characterized by the presence of super conjugation of π electrons in their chemical structures, enabling the absorption of low‐energy photons (long wavelengths) to activate NIR‐RDRP, including, NIR‐RAFT polymerization, NIR‐ATRP, and NIR light‐induced RCMP (NIR‐RCMP). Various chromophores have been exploited as PCs (**Figure** **8** and **Table** **2**), including bacteriochlorins,^[^ ^32^, ^33^ ^]^ porphyrins,^[^ ^8^ ^]^ metal phthalocyanines,^[^ ^19^, ^23^, ^25^, ^33^ ^]^ iron dicarbonyl dimer,^[^ ^20^, ^33^ ^]^ polymethines,^[^ ^25a,c,d^ ^]^ carbocyanine,^[^ ^25^, ^32^ ^]^ and carbonyl solvents.^[^ ^25j,k^ ^]^ In addition to these organic molecules, nanocrystals,^[^ ^22^, ^25^, ^49^ ^]^ and nanohybrids^[^ ^22c^ ^]^ have also been employed as PCs in NIR‐RAFT polymerization. In contrast to UCNP‐mediated systems which require CW lasers for activation, the PC‐mediated NIR‐RDRP operates via a single‐photon process. This means that specific light sources are not necessary, making the process more accessible and cost‐effective. NIR LEDs are commonly used as the light source in these systems due to their affordability, safety, and ease of use.

**Table 2:** Summary of PC‐mediated NIR‐RDRP systems

# and refs.	PC	RDRP mechanism	Light source and intensity (*I*)	Polymerization performance: conversion (*α*) and dispersity (*Đ*)	Monomer versatility	Solvents
1^[^ ^25a^ ^]^	Polymethine	ATRP (Figure 9A)	790 nm LED (*I* = 100 mW cm^−2^)	Methyl methacrylate (MMA): *α* = 44.3% in 24 h *Đ* = 1.15	Methacrylate and styrene	DMF
2^[^ ^25b^ ^]^	TTPDPT	ATRP (Figure 9C)	830–890 nm CW laser (*I* = 35 W cm^−2^)	MMA: *α* ≈ 50% in 10 h *Đ* ≈ 1.25	Methacrylate	CH_2_Cl_2_
3^[^ ^32a^ ^]^	BChl a	RAFT (Figure 10A)	780 nm LED (*I* = 104.9 mW cm^−2^)	MMA: *α* = 74% in 20 h *Đ* = 1.09	Acrylate and methacrylate	DMSO
4^[^ ^33a^ ^]^	RTPP	RAFT (Figure 10A)	740 nm LED (*I* = 66 mW cm^−2^)	MMA: *α* = 89% in 12 h *Đ* = 1.13	Acrylate and methacrylate	DMSO
5^[^ ^50^ ^]^	FBC	RAFT (Figure 10A)	740 nm LED (*I* = 66 mW cm^−2^)	Trifluoromethyl methacrylate: *α* = 78% in 24 h *Đ* = 1.19	Methacrylate	DMSO
6^[^ ^25f^ ^]^	AlPc	RAFT (Ref. [25f])	780 nm LED (*I* = 6.2 mW cm^−2^)	Methyl acrylate (MA): 66% in 6 h *Đ* = 1.10	Acrylate, acrylamide, and methacrylate	NMP
7^[^ ^51^ ^]^	AlNc	RAFT (Figure 11A)	780 nm LED (*I* = 20 mW cm^−2^)	MA: 76% in 1.5 h *Đ* = 1.12	Acrylate, acrylamide, and methacrylate	DMSO
8^[^ ^52^ ^]^	ZnTtBNc	RAFT (Figure 10A)	780 nm LED (*I* = 35 mW cm^−2^)	MMA: *α* = 69% in 5 h *Đ* = 1.19	Acrylate and methacrylate	DMSO
9^[^ ^8^ ^]^	SA‐TCPP	RAFT (Figure 12A)	850 nm LED (*I* = 4 mW cm^−2^)	*N*,*N*‐Dimethylacrylamide (DMA): *α* = 53% in 66 h *Đ* ≈ 1.1	Acrylamide	Water
10^[^ ^17c^ ^]^	ZnPcS_4_^−^	RAFT (Figure 13D)	730 nm LED (*I* = 60 mW cm^−2^)	DMA: *α* = 65% in 3 h *Đ* = 1.09	Acrylate, acrylamide, and methacrylate	Water
11^[^ ^25g^ ^]^	*β*‐TS‐Zn‐M1	RAFT (Figure 11A)	730 nm LED (*I* = 75.7 mW cm^−2^)	DMA: *α* = 90% in 2 h *Đ* = 1.09	Acrylate and acrylamide	Water
12^[^ ^19^ ^]^	PPc‐MPEG	RAFT (Figure 10A)	760 nm LED (*I* = 20 mW cm^−2^)	DMA: *α* = 84.6% in 4 h *Đ* = 1.05	Acrylamide	Water
13^[^ ^33b^ ^]^	Fe(Cp)_2_(CO)_4_	RAFT (Figure 14A)	788 nm LED (*I* = 14 mW cm^−2^)	Isobutyl vinyl ether: *α* = 92.1% in 1 h *Đ* = 1.07	Vinyl ether, acrylate, acrylamide, methacrylate, and styrene	No
14^[^ ^20^ ^]^	Fe(Cp)_2_(CO)_4_	RAFT (Figure 14B)	788 nm LED (*I* = 20.6 mW cm^−2^)	Isobutyl vinyl ether: *α* = 99.9% in 20 min *Đ* = 1.07	Vinyl ether	Ethyl acetate
15^[^ ^22c^ ^]^	CsPbI_3_@PCN‐222	RAFT (Figure 15A)	850 nm LED (*I* = 50 mW cm^−2^)	MMA: *α* = 80% in 4 h *Đ* = 1.07	Acrylate, acrylamide, methacrylate, styrene, vinyl acetate, *N*‐vinylpyrrolidone	DMSO, DMF, water, and toluene.
16^[^ ^49^ ^]^	Ag_3_PO_4_	RAFT (Figure 16E)	940 nm LED (*I* = 16 mW cm^−2^)	Benzyl acrylate: *α* = 89.6% in 14.5 h *Đ* = 1.22	Acrylate	DMSO
17^[^ ^25h^ ^]^	Au nanocrystals	RAFT^[^ ^25h^ ^]^	740 nm LED (*I* = 0.7 mW cm^−2^)	MMA: *α* = 51.5% in 72 h *Đ* = 1.16	Acrylate, methacrylate, and styrene	DMSO, DMF, MeCN, and THF
18^[^ ^25i^ ^]^	Au/graphitic carbon nitride	RAFT^[^ ^25i^ ^]^	740 nm LED (*I* = 0.7 mW cm^−2^)	MMA: *α* = 18.1% in 20 h *Đ* = 1.31	Methacrylate	DMSO
19^[^ ^53^ ^]^	C3‐DCl	RCMP (Figure 17C)	700 ± 50 nm xenon lamp with an optical filter	MMA: *α* = 43% in 24 h *Đ* = 1.19	Methacrylate	Diglyme
20^[^ ^25l^ ^]^	EI	RCMP (Figure 17C)	800 nm LED (*I* = 11 mW cm^−2^)	Poly(ethylene glycol) methyl ether methacrylate (POEGMA): *α* = 37.4% in 12 h *Đ* = 1.14	Methacrylate	Water
21^[^ ^25j^ ^]^	“Carbonyl” solvent	RCMP (Figure 18)	730 nm LED (*I* = 66.3 mW cm^−2^)	MMA: *α* = 97.9% in 18 h *Đ* = 1.19	Methacrylate	DMI, TMU, DMPU, DMAc, and NMP
22^[^ ^54^ ^]^	NTCDA	RCMP^[^ ^54^ ^]^	740 nm LED (*I* = 70.58 mW cm^−2^)	MMA: *α* = 95.8% in 4 h *Đ* = 1.18	Methacrylate	DMI, TMU, DMPU, DMAc, and NMP

#### 2.2.1 PC‐Mediated NIR‐ATRP

**Figure 9:**
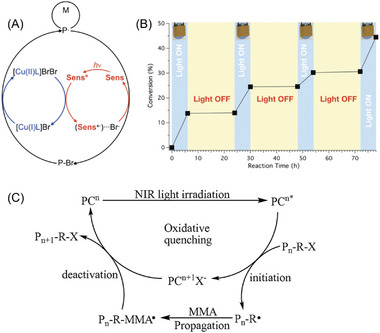
A) Proposed mechanism of NIR‐ATRP using a polymethine as a sensitizer (Sens). B) Demonstration of temporal control of NIR‐ATRP process. (A,B) Reproduced with permission.^[^ ^25a^ ^]^ Copyright 2018, John Wiley & Sons. C) Mechanism of photoinduced metal‐free ATRP of MMA using TTPDPT as photocatalyst under NIR light irradiation. Reproduced with permission.^[^ ^25b^ ^]^ Copyright 2018, Springer Nature.

In a conventional photo‐ATRP system, UV or blue light is usually required to successfully reduce the copper(II) catalyst to copper(I).^[^ ^55^ ^]^ However, this poses a challenge when polymerizing UV‐absorbing monomers via photo‐ATRP, as these monomers hinder the transmission of UV to PCs. As a result, the reduction of the copper(II) catalyst to its active copper(I) state becomes inefficient, leading to difficulties in achieving controlled polymerization. To overcome this challenge, Strehmel's group developed an NIR‐ATRP system sensitized by a polymethine (Figure 8, #1; Table 2, #1) in the presence of ppm Cu^II^/TPMA as the catalyst and α‐bromophenylacetate as the alkyl halide initiator.^[^ ^25a^ ^]^ The polymethine has a typical absorption on the NIR region (*λ* ≈ 790 nm) with a high molecular extinction coefficient (263 000 m ^−1^ cm^−1^ in DMF), and it was utilized as a sensitizer (Sens) to mediate ATRP. In the proposed mechanism, Sens is activated to its excited states (Sens*) under irradiation. An electron transfer process from Sens* to [Cu(II)L]BrBr is followed, generating [Cu(I)L]Br and anionic radical (Sens^•+^)Br^−^. Similar to conventional ATRP, P_n_–Br is activated by [Cu(I)L]Br, which leads to the generation of initiating radicals P_n_^•^ during polymerization, as illustrated in **Figure** **9**A. Meanwhile, the photocatalytic cycle is completed by deactivating propagating radicals by (Sens^•+^)Br^−^, regenerating P_n_–Br and Sens. In the NIR‐ATRP system, the controlled character was experimentally demonstrated by observing a linear relationship between ln([M]_0_/[M]_t_) at different exposure times. Furthermore, this approach offers temporal control over the photopolymerization process, allowing precise manipulation of the polymerization reaction (Figure 9B).^[^ ^56^ ^]^ In 2020, the same NIR‐ATRP was successfully employed for the photopolymerization of monomers containing UV‐absorbing moieties. This was also achieved by using polymethine as PC (Figure 8, #1).^[^ ^25c^ ^]^ The resulting polymers incorporating UV‐absorbing groups can be utilized in various applications such as coatings^[^ ^57^ ^]^ and filter materials.^[^ ^58^ ^]^ In 2021, Strehmel and co‐workers modified the NIR‐ATRP system by employing Fe(III) as the catalyst instead of Cu(II). This addressed the challenge of oxygen inhibition in radical polymerization, allowing for well‐defined polymer synthesis under aerobic conditions.^[^ ^25d^ ^]^

To address the issue of metal contamination associated with ATRP, Wang et al. reported a metal‐free NIR‐ATRP system in 2018. They utilized pentarylenebis(dicarboximide) dye, named (1,6,13,18‐tetra(4‐(2,3,3‐trimethylbut‐2‐yl)phenoxy)‐*N,N′*‐(2,6‐diisopropylphenyl)‐pentarylene‐3,4,15,16 tetracarboxidiimide) (TTPDPT) as a PC (Table 2, #2).^[^ ^25b^ ^]^ TTPDPT possesses an extended conjugation structure of π electrons (Figure 8, #2), enabling absorption in the NIR region of the electromagnetic spectrum (800–900 nm).^[^ ^59^ ^]^ The proposed mechanism (Figure 9C) for this metal‐free NIR‐ATRP system involves the photoactivation of the PC by NIR irradiation, promoting its transition to an excited state (PC*). Subsequently, an electron transfer occurs from PC* to the dormant species, P_n_–X. As a result, cationic radical formation of PC (PC^•+^/X^−^) and initiating species P_n_^•^ are generated. This system was demonstrated to be efficient for the polymerization of MMA. Concurrently, P_n_–MMA^•^ is deactivated by PC^•+^/X^−^ to regenerate P_n_–MMA–X and PC, making the catalytic cycle complete.

#### 2.2.2 PC‐Mediated NIR‐RAFT Polymerization

**Figure 10:**
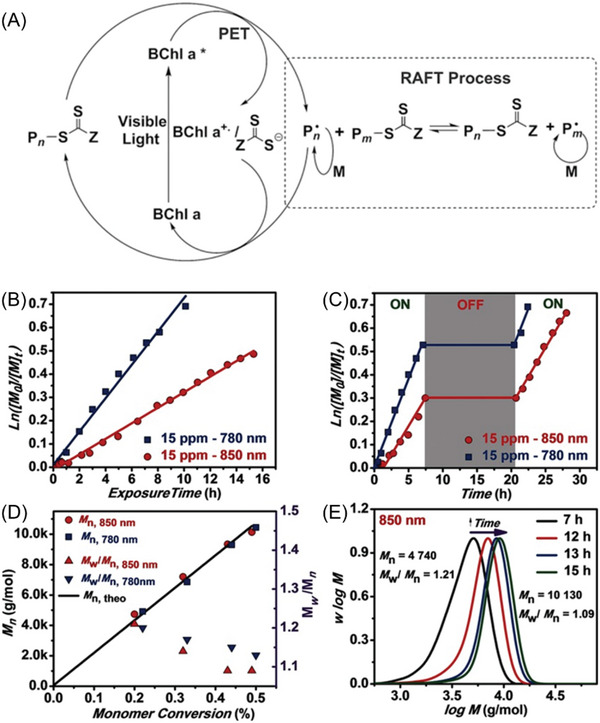
A) Proposed mechanism for PET‐RAFT polymerization using BChl a as the photocatalyst. B) ln([M]_0_/[M]_t_) versus light exposure time under 850 nm (red circles) and 780 nm (blue squares) irradiation. C) Demonstration of temporal control on PET‐RAFT polymerization under 780 and 850 nm wavelengths. D) Dependence of *M*_n_ on the conversion under NIR irradiation. E) Molecular weight distributions after exposure to NIR irradiation for different periods of time. Reproduced with permission.^[^ ^32a^ ^]^ Copyright 2016, John Wiley & Sons.

In 2015, Boyer and co‐workers reported a pioneering study where they used bacteriochlorophyll a (BChl a) as a PC (Figure 8, #3) to activate a PET‐RAFT polymerization under NIR light irradiation (Table 2, #3).^[^ ^32a^ ^]^ The photocatalytic mechanism involves an oxidative quenching pathway through the PET‐RAFT process (**Figure** **10**A). In this system, when exposed to light, the excited PC transfers an electron to the RAFT agents, leading to the formation of a PC cationic radical (PC^•+^) and initiating radical/residual RAFT anionic radical (P_n_^•^/RAFT agent^•−^). Subsequently, chain propagation occurs in the presence of P_n_^•^, and P_n_^•^ can be efficiently deactivated to a dormant chain or chain transferred. The polymerization can also be deactivated by back electron transfer from P_n_^•^/RAFT agent^•−^ and PC^•+^, to regenerate the dormant P_n_‐RAFT agent and PC. Experimental results showed successful polymerizations under NIR light at 780 and 850 nm, with a linear relationship between ln([M]_0_/[M]_t_) and the exposure time (Figure 10B). The system demonstrated excellent temporal control, presenting no monomer conversion in darkness (Figure 10C). As the monomer conversion increased, the dispersity index (*Đ*) decreased from 1.2 to 1.1 (Figure 10D), suggesting a controlled polymerization mechanism. The MWDs of the polymers shifted toward higher molecular weights as monomer conversion increased (Figure 10E). Taking advantage of the enhanced penetration of NIR wavelengths, the synthesis of well‐defined polymethacrylates through various thicknesses of opaque materials (paper sheets, in this case), was demonstrated. However, the high cost of commercial BChl a, which is primarily obtained from phototrophic bacteria, limited its broader applications.

In subsequent work by Zhang's group, a synthetic bacteriochlorin PC was prepared by reducing tetraphenyl porphyrin (TPP) for the mediation of PET‐RAFT polymerization.^[^ ^33a^ ^]^ The reduced tetraphenyl porphyrin (RTPP) displays a shifted absorption at longer wavelengths (700–765 nm) (Figure 8, #4; Table 2, #4) compared to TPP, thanks to the reduced energy space between the highest occupied molecular orbital and the lowest unoccupied molecular orbital.^[^ ^60^ ^]^ In contrast to the BChl a, the system demonstrated versatility toward the polymerization of acrylates and methacrylates. Owing to the high penetration of 740 nm light, controlled radical polymerization was successfully achieved through various biological barriers, including pig skin and chicken skin. In their following work, the authors modified RTPP with Fluorine atoms (Figure 8, #5; Table 2, #5) to yield fluorophenyl bacteriochlorin (FBC), intending to enhance the photostability of RTPP.^[^ ^61^ ^]^ FBC enabled the PET‐RAFT polymerization of semifluorinated methacrylic monomers under NIR light.^[^ ^50^ ^]^ The system was employed for the preparation of polymeric nanoparticles and the fabrication of polymer brushes on wafers.

Phthalocyanines and their metal complexes have also been explored as PCs under long wavelengths (Figure 8, #6–8 and #10–12). Besides their capacity to mediate PET‐RAFT systems, these PCs were reported with the ability to react with solvent or peroxide to generate initiating radical species to efficiently mediate photo‐RAFT polymerization. In 2016, Boyer and colleagues reported a photoinitiation system utilizing various metal phthalocyanines (MPcs), including zinc phthalocyanine (ZnPc), magnesium phthalocyanine (MgPc), and aluminum phthalocyanine chloride (AlPc), for RAFT polymerization under 780 nm light irradiation (Figure 8, #6; Table 2, #6).^[^ ^25f^ ^]^ Interestingly, successful photo‐RAFT polymerization was only observed in *N*‐methyl‐2‐pyrrolidone (NMP) solvent, while no polymerization occurred in other solvents, such as DMAc, DMF, and DMSO. This was attributed to the electron‐donating ability of NMP. The proposed mechanism proposes that the excited state of PC leads to oxidation of the NMP followed by deprotonation and rearrangement of NMP yielding an initiating radical.^[^ ^62^ ^]^ Although the mechanism requires more investigation, this NIR‐RAFT system displays good oxygen tolerance, enabling the preparation of well‐defined polymers in an open‐air environment. This MPcs‐catalyzed NIR‐RAFT system was utilized by Hu's group for surface modification of poly(vinyl alcohol) (PVA) to enhance antifouling properties.^[^ ^33c^ ^]^ Under NIR irradiation, carboxybetaine methacrylate (CBMA) polymer brushes were successfully grafted onto PVA hydrogels via photo‐RAFT polymerization.

**Figure 11:**
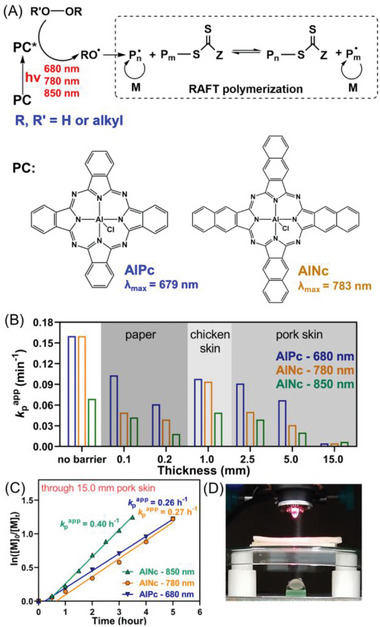
A) Long wavelength photosensitization of (hydro)peroxides to generate radicals under 680, 780, and 850 nm wavelengths mediated by aluminum phthalocyanine (AlPc) and aluminum naphthalocyanine (AlNc). B) Comparison of apparent propagation rate coefficients (*k*_p_^app^) of photo‐RAFT polymerizations of MA mediated by AlPc and AlNc under far‐red and NIR light through barriers. C) Kinetic comparison of RAFT photopolymerizations performed through 15.0 mm thick pig skin. D) Photo detailing the experimental setup used for polymerizing through 5.0 mm pig skin. Reproduced with permission.^[^ ^51^ ^]^ Copyright 2020, John Wiley & Sons.

Our group also utilized aluminum naphthalocyanine (AlNc) and AlPc as PCs (Figure 8, #7; Table 2, #7) to activate photo‐RAFT polymerization under red and NIR light irradiation.^[^ ^51^ ^]^ In contrast to previous NIR light‐induced PET‐RAFT systems, where the generation of radicals is by the dissociation of RAFT agent, the generation of initiating radicals was achieved through the photosensitization of various (hydro)peroxides (**Figure** **11**A).^[^ ^63^ ^]^ Under NIR light irradiation (*λ*_max_ = 780 nm; *I* = 100 mW cm^−2^), a fast polymerization rate (*k*_p_^app^ = 0.15 min^−1^) was achieved using AlNc as PC, leading to over 80% monomer conversion within 10 min. The high penetration of NIR light allowed for highly efficient photopolymerization even in the presence of thick barriers, such as 5.0 mm thick pig skins, achieving a rate of *k*_p_^app^ = 0.06 min^−1^ (Figure 11B,D). Moreover, controlled radical polymerization was successfully achieved through pig skin barriers with a thickness of 15.0 mm, resulting in over 70% monomer conversion within 4 h (Figure 11C). In 2022, the same group utilized zinc 2,11,20,29‐tetra‐*tert*‐butyl‐2,3‐naphthalocyanine (ZnTtBNc) as an efficient PC (Figure 8, #8; Table 2, #8) to activate PET‐RAFT polymerization of acrylates and methacrylates via an oxidative quenching pathway (Figure 10A).^[^ ^52^ ^]^ Interestingly, the lower redox potential of ZnTtBNc compared to previously explored PCs leads to the unique selectivity of photoactivation of trithiocarbonate (TTC) RAFT agent. Specifically, TTC with a tertiary carbon R group could be activated by excited ZnTtBNc leading to successful polymerization of acrylates, while no monomer conversion was observed in the presence of TTC with a secondary R group. By employing density functional theory calculations in combination with experimental studies, new mechanistic insights into the factors governing the PET‐RAFT mechanism and the selectivity of ZnTtBNc toward tertiary carbon TTCs were revealed. Additionally, a moderate increase in reaction temperature (≈15 °C) was found to overcome the photoactivation barrier of TTC with secondary R groups when using ZnTtBNc as PC, enabling successful PET‐RAFT polymerization of acrylates under NIR irradiation.

**Figure 12:**
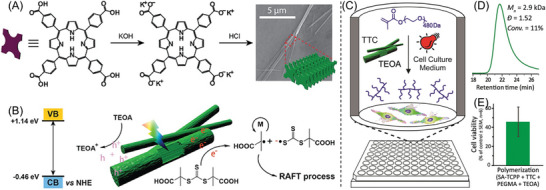
A) The synthetic strategy for rod‐like SA‐TCPP. B) Schematic mechanism of the photocatalytic PET‐RAFT polymerizations by SA‐TCPP. C) Microliter‐scale PET‐RAFT polymerization of PEGMA_480_ in a 96‐well plate containing fibroblast cells, 45 min of red‐light irradiation. D) GPC trace of poly(PEGMA). E) Cell viability following PET‐RAFT polymerization. Reproduced with permission.^[^ ^8^ ^]^ Copyright 2020, John Wiley & Sons.

Although NIR‐RAFT systems had been well‐established in organic solvents, such as DMSO, DMF, and toluene, there was no aqueous system until 2020. Using aqueous media instead of organic solvents for polymerization provides economic and environmental benefits and opens potential bioapplications. Qiao's group developed the first aqueous NIR‐RAFT system (Table 2, #9) by utilizing a self‐assembled carboxylated porphyrin (SA‐TCPP)^[^ ^64^ ^]^ as PC (Figure 8, #9; **Figure** **12**A).^[^ ^8^ ^]^ SA‐TCPP displays a broad absorbance spectrum from 300 to 950 nm, exhibiting the ability of a broadband PC to mediate PET‐RAFT polymerization under various wavelength irradiation, including blue, green, red, and NIR light. The proposed mechanism for this PET‐RAFT process involves a reductive quenching pathway of PC, where a tertiary amine interacts with the PC (Figure 12B). When exposed to light, the PC is excited and acquires an electron from triethanolamine (TEOA), leading to the formation of anionic radical (PC^•−^) and cationic radical (TEOA^•+^). Subsequently, the RAFT agent gains an electron from PC^•−^ via an electron transfer, resulting in its fragmentation into an initiating species and regeneration of PC. Although the photopolymerization process exhibited a relatively slow rate, with 53% monomer conversion achieved in 66 h under NIR light irradiation, it was demonstrated that well‐defined polymers could be successfully synthesized even through nontransparent barriers like paper sheets. A notable aspect of this work is the possibility of achieving PET‐RAFT polymerization in the presence of mammalian fibroblast cells in a 96‐well plate (Figure 12C,D). The outcome of these experiments was the synthesis of well‐defined polymers with relatively high cell viability (46%), as shown in Figure 12E.

In a recent groundbreaking achievement, Cai and colleagues pioneered the development of a porphyrin‐containing conjugated microporous polymers (CMPs) using Sonogashira–Hagihara polycondensation, where 5,10,15,20‐tetra(4‐ethynylphenyl)‐21H,23H‐porphyrin and 1,4‐diiodobenzene were polymerized onto SiO_2_ microspheres and subsequently the SiO_2_ templates were removed.^[^ ^65^ ^]^ These CMPs were effectively harnessed as heterogeneous photosensitizers to induce aqueous PET‐RAFT polymerization of diverse monomers when exposed to 740 nm NIR light. Extensive experimentation has revealed that the catalytic efficiency in photopolymerization is profoundly influenced by the structure of CMPs. What distinguishes these nanocomposites are their remarkable ease of separation and purification from the reaction mixture. Their catalytic efficiency can be maintained over several recycling cycles, enabling multiple polymerizations.

**Figure 13:**
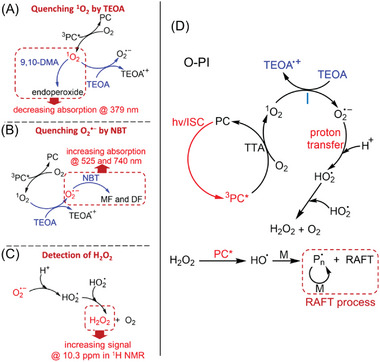
Experimental evidence of an oxygen‐mediated photoinitiation system in the presence of TEOA and oxygen. A) Experiments of quenching singlet oxygen (^1^O_2_) in the presence of 9,10‐dimethylanthracene (9,10‐DMA) with different ratios of TEOA using a stoichiometry. B) Experiments of quenching superoxide (O_2_^•−^) in the presence of nitrotetrazolium blue chloride (NBT) with different ratios of TEOA using a stoichiometry. C) The formation of H_2_O_2_ as indicated by ^1^H NMR spectra (d_6_‐DMSO) with increasing irradiation time in the presence of ZnPcS_4_^−^ and different concentrations of TEOA. D) Photo‐RAFT polymerization via an oxygen‐mediated photoinitiation (O‐PI) system. Reproduced with permission.^[^ ^17c^ ^]^ Copyright 2022, The Royal Society of Chemistry.

Later, other PCs, such as phthalocyanines and their metal complexes, have been utilized for aqueous photo‐RAFT polymerization under NIR light (Figure 8, #10–12). In 2022, Wu et al. developed an NIR‐RAFT system in water (Figure 8, #10; Table 2, #10) utilizing tetrasulfonated zinc phthalocyanine (ZnPcS_4_^−^) as water‐soluble PC in the presence of TEOA.^[^ ^17c^ ^]^ This system demonstrated living characteristics and high efficiency in the photopolymerization of various monomers. Interestingly, it was observed that the polymerization rate was much faster in the presence of oxygen, indicating a peculiar photochemical reaction pathway. To investigate this special photo‐RAFT mechanism, several characterizations were conducted, including quenching experiments for reactive oxygen species (ROS) and ^1^H NMR spectroscopy (**Figure** **13**A–C). As a result, an oxygen‐mediated photoinitiation (O‐PI) pathway (Figure 13D) was proposed. Under light irradiation, the ground state PC is excited to ^3^PC*. TTA occurs between ^3^PC* and O_2_, generating singlet oxygen (^1^O_2_). ^1^O_2_ reacts with the reducing agent TEOA via an electron transfer process, resulting in the formation of TEOA^•+^ and superoxide (O_2_^•−^). Subsequently, the protonation of O_2_^•−^ leads to the formation of hydroperoxyl radical (HO_2_^•^),^[^ ^66^ ^]^ which is regarded as a precursor of H_2_O_2_.^[^ ^67^ ^]^ Consequently, H_2_O_2_ is photosensitized by excited PC* under light irradiation to generate hydroxyl radicals, initiating RAFT polymerization.^[^ ^63^ ^]^ This system was the first aqueous NIR‐RAFT polymerization displaying excellent oxygen tolerance with fast polymerization rates. Taking advantage of these properties, this NIR‐RAFT system was successfully applied in the synthesis of polymeric nanoparticles, (see Section 3.5).

Inspired by the activation of H_2_O_2_ to initiate RAFT polymerization, Cheng's group synthesized a water‐soluble zinc phthalocyanine (β‐TS‐Zn‐1) by functionalization of aromatic rings with tri(ethylene glycol) groups for photo‐RAFT polymerization in the presence of H_2_O_2_ under NIR irradiation.^[^ ^25g^ ^]^ Moreover, a type of novel polymerizable acrylate containing zinc phthalocyanine units (β‐TS‐Zn‐M1) was successfully synthesized from the reaction between β‐TS‐Zn‐1 and acryloyl chloride (Figure 8, #11; Table 2, #11). In comparison with PCs, these PCs are loaded on polymer chains, facilitating the procedure of post‐treatment. Besides the strategy to enhance the solubility of PC for homogenous photochemical reactions, an alternative way is to develop suspended catalysts for a heterogenous photopolymerization in the aqueous solution.^[^ ^68^ ^]^ In 2023, Cai and co‐workers developed a novel poly(ethylene glycol) methyl ether (MPEG)‐linked polyphthalocyanine (PPc‐MPEG), which can generate a stable colloidal suspension in water (Figure 8, #12).^[^ ^19^ ^]^ Owing to this novel water‐suspended PPc‐MPEG possessing absorption of long wavelengths, it is successfully exploited as PC to induce heterogenous PET‐RAFT polymerization under NIR irradiation (Table 2, #12). Utilization of suspended PPc‐MPEG for heterogeneous NIR‐RAFT polymerization benefits the recyclization of PCs. This strategy has been successfully applied to membrane reactors for upscaling the production of well‐defined polymers.

**Figure 14:**
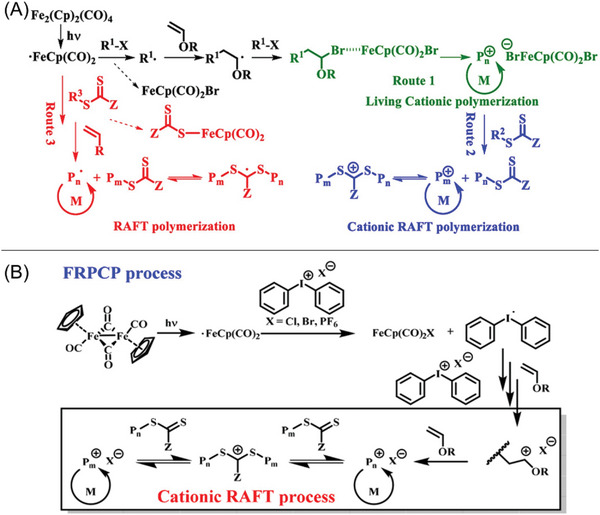
A) Proposed mechanism of the photopolymerization mediated by Fe_2_(Cp)_2_(CO)_4_ and via RAFT cationic polymerization (Route 1 and Route 2) and RAFT radical polymerization (Route 3). B) Proposed mechanism for photoinduced free radical‐promoted cationic RAFT polymerization mediated by Fe_2_(Cp)_2_(CO)_4_ and diphenyliodonium salt. A) Reproduced with permission.^[^ ^33b^ ^]^ Copyright 2020, American Chemical Society. B) Reproduced with permission.^[^ ^20^ ^]^ Copyright 2021, American Chemical Society.

Cyclopentadienyl iron dicarbonyl dimer (Fe_2_(Cp)_2_(CO)_4_) was developed by Zhu and co‐workers as a new photosensitizer to induce NIR‐RAFT polymerization in bulk (Figure 8, #13; Table 2, #13).^[^ ^33b^ ^]^ Under NIR irradiation, Fe_2_(Cp)_2_(CO)_4_ was demonstrated to generate initiating radicals, inducing both RAFT radical polymerization of acrylates and RAFT cationic polymerization of vinyl ethers successfully. Well‐defined poly(vinyl ether)s and polyacrylates can be prepared via this system. In the proposed mechanism of RAFT radical polymerization, homolytic photolysis of the Fe─Fe bond occurs under light irradiation. These fragmented species can react with RAFT agents to generate iron‐dithiocabonate complexes and initiating radicals, leading to successful RAFT radical polymerization (**Figure** **14**A, Route 3).^[^ ^69^ ^]^ In addition to the radical polymerization, the dissociation of iron‐dithiocabonate complexes in the presence of vinyl ether and halide compounds can lead to a cationic polymerization pathway (Figure 14A, Route 1).^[^ ^70^ ^]^ In the cationic pathway, the generated Fe radical reacts with an organic halide via the halide abstraction process, resulting in the formation of FeCp(CO)_2_Br and cationic propagating species. By adding RAFT agents, cationic RAFT polymerization can be induced successfully (Figure 14A, Route 2). Interestingly, excellent penetration of NIR light enables the synthesis of various types of polymers through thick barriers in a controlled manner. Moreover, this system was successfully applied in 3D printing to fabricate materials with different thicknesses. In 2021, the same group developed a new system by combining Fe(Cp)_2_(CO)_4_ with diphenyliodonium salt (Table 2, #14), which was demonstrated with a significantly higher efficiency under NIR light than the previous system (Table 2, #14).^[^ ^20^ ^]^ In their proposed mechanism, Fe(Cp)_2_(CO)_4_ is first decomposed under light, followed by the reduction and decomposition of the onium salt.^[^ ^71^ ^]^ These generated radicals further react with the monomers and onium salts to form initiating cations (Figure 14B), which was reported by earlier contributions.^[^ ^72^ ^]^

**Figure 15:**
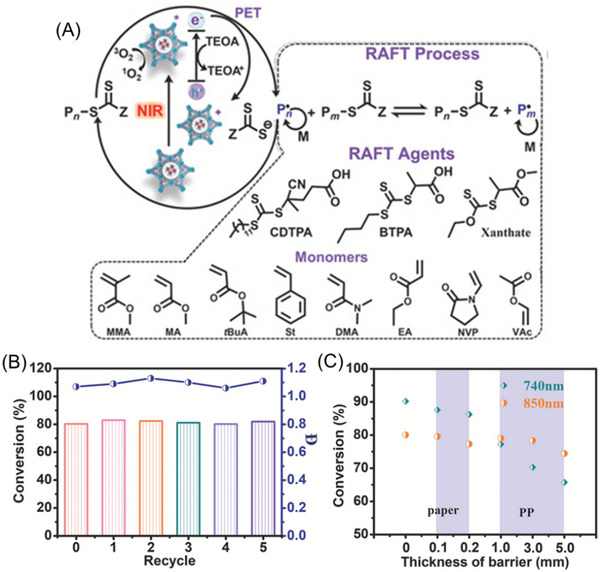
A) The proposed mechanism of PET‐RAFT polymerization catalyzed by CsPbI_3_@PCN‐222 under NIR light using various RAFT agents and monomers. B) Cyclic stability test of CsPbI3@PCN‐222(20%) photocatalyst under 850 nm light and C) dependence of MMA monomer conversion on the thickness of the barrier (paper and PP) in PET‐RAFT polymerization of MMA with DMSO as solvent in air for 4 h. Adapted with permission.^[^ ^22c^ ^]^ Copyright 2022, John Wiley & Sons.

To address the recycling issue of these PCs, Lü and co‐workers developed a heterogenous all‐inorganic halide perovskite encapsulated in Zr‐based metal‐organic frameworks (Zr‐MOF) (Table 2, #15; **Figure** **15**A).^[^ ^22c^ ^]^ This binary nanohybrid PCs, composed of CsPbI_3_ and porphyrinic Zr‐MOF PCN‐222, displayed a high photocatalytic activity to activate a reductive PET‐RAFT polymerization without deoxygenation under red to NIR light irradiation.^[^ ^73^ ^]^ Various monomers, including acrylates, acrylamides, methacrylates, styrene, vinyl ether, and *N*‐vinyl pyrrolidinone, were successfully synthesized in controlled manners via this process, demonstrating its versatility. Furthermore, CsPbI_3_@PCN‐222 PC was easily separated from the polymerization mixtures and reused in 5 cycles without a significant reduction in catalytic performance (Figure 15B). Furthermore, the system's high efficiency in achieving rapid polymerization rates under long‐wavelength irradiation was demonstrated by conducting PET‐RAFT polymerization with various thicknesses of paper and polypropylene (PP) boards placed between the reaction mixture and the NIR light source (Figure 15C).

**Figure 16:**
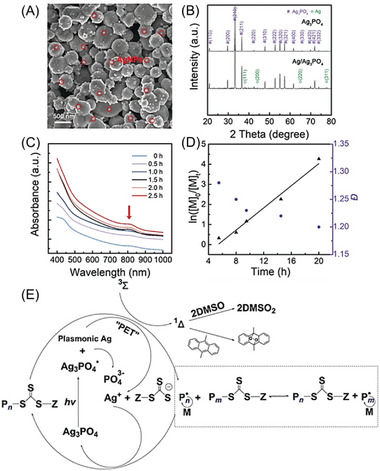
A) SEM of AgNPs generated on Ag_3_PO_4_ surfaces. B) XRD patterns of Ag_3_PO_4_ and Ag/Ag_3_PO_4_ mixture from the reaction. C) UV/vis/NIR spectra of Ag_3_PO_4_ and the plasmonic AgNPs generated in the polymerization system with increasing irradiation time. D) Kinetic study of Ag_3_PO_4_‐catalyzed RAFT polymerization of BzA under 780 nm LED irradiation the dispersity (*Đ*). E) Proposed mechanism of Ag_3_PO_4_‐catalyzed RAFT polymerization. Adapted with permission.^[^ ^49^ ^]^ Copyright 2019, John Wiley & Sons.

The localized surface plasmon resonance (LSPR)^[^ ^74^ ^]^ strategies were first utilized to harvest broadband light for PET‐RAFT polymerization (Table 2, #16) in 2019, which was reported by Matyjaszewski and co‐workers.^[^ ^49^ ^]^ The photocatalytic activity is enhanced via the in situ formation of plasmonic Ag nanoparticles (AgNPs) on the surface of Ag_3_PO_4_ photocatalysts, which was evidenced by SEM (**Figure** **16**A) and XRD characterizations (Figure 16B).^[^ ^75^ ^]^ In this process, an increasing LSPR absorption was observed in visible and NIR light regions (Figure 16C), enabling efficient polymerization of benzyl acrylate (BzA) in a controlled manner under 780 nm light (Figure 16D). In the proposed mechanism (Figure 16E), Ag_3_PO_4_ is excited under light irradiation, which is followed by self‐photoreduction to generate metallic AgNPs on the surface of Ag_3_PO_4_. Owing to the LSPR effect promoting the charge separation in Ag_3_PO_4_, a photoinduced electron transfer is favorable from generated plasmonic AgNPs to RAFT agents.^[^ ^76^ ^]^ As a result, silver ions are regenerated, and RAFT agents play roles both as initiators and chain transfer agents in radical polymerization. The first case of LSPR‐enhanced PET‐RAFT polymerization under visible or NIR light was introduced in this study, offering great potential for advanced macromolecular synthesis. In 2021, Pang and co‐workers exploited Au nanocrystals as PCs for PET‐RAFT polymerization via LSPR (Table 2, #17).^[^ ^25h^ ^]^ Au nanocrystals with various morphologies, including nanospheres and nanorods were synthesized. These nanocrystals can harvest the energy from visible light to NIR light for PET‐RAFT polymerization of MMA. In 2022, the same group developed a PET‐RAFT process mediated by a series of Au/graphitic carbon nitride (g‐C_3_N_4_) composite photocatalysts (Table 2, #18).^[^ ^25i^ ^]^ The Schottky barrier formation improved the migration of carriers by reducing the rate of charge recombination,^[^ ^77^ ^]^ resulting in the superior performance of Au/g‐C_3_N_4_ nanohybrids compared to Au nanoparticles and g‐C_3_N_4_.

#### 2.2.3 PC‐Mediated NIR‐RCMP

**Figure 17:**
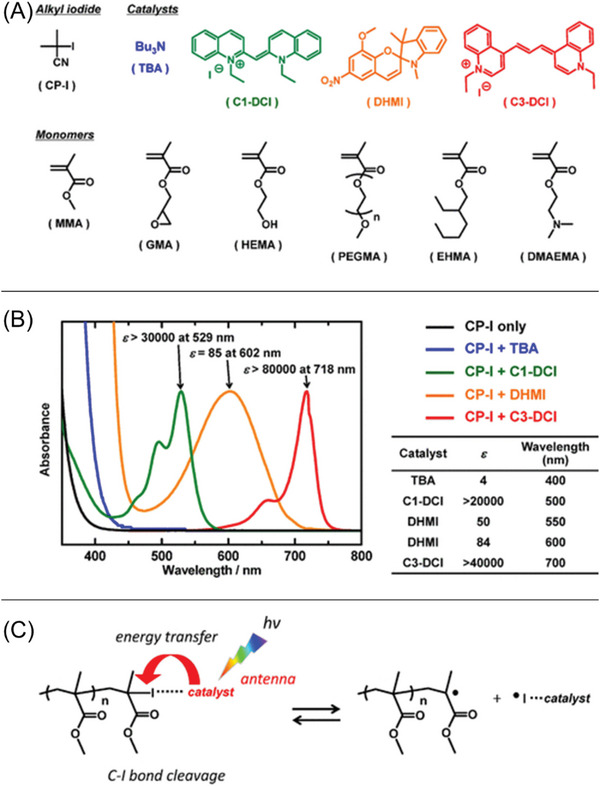
A) Structures of an alkyl iodide, catalysts, and monomers used in this photo‐RCMP system. B) UV–vis–NIR spectra of CP‐I and the mixtures of CP‐I. Extinction coefficients (*ε*) for mixtures of CP‐I in the presence of cocatalysts. C) Proposed mechanism for photo‐RCMP via C─I bond cleavage of polymer iodide and subsequent energy transfer under irradiation. Reproduced with permission.^[^ ^32b^ ^]^ Copyright 2015, American Chemical Society.

Goto and co‐workers introduced a metal‐free photo‐RDRP (Table 2, #19), called RCMP.^[^ ^53^, ^78^ ^]^ This approach utilizes an alkyl iodide (CP‐I) as a control agent for RDRP and amines as catalysts (**Figure** **17**A). One notable advantage of RCMP that was discovered in 2015 is its ability to be initiated by a broad range of wavelengths (Figure 17B), when suitable amine catalysts are employed, spanning from blue to NIR light (350–750 nm).^[^ ^32b^ ^]^ Various polymethacrylates and polystyrene (Figure 17A) were successfully synthesized in a controlled manner via this process. In the proposed mechanism, light irradiation triggers the cleavage of the C─I bond within the complex (P_n_–I–catalyst) generating P_n_^•^ and ^•^I–catalyst complex (Figure 17C). Chain propagation occurs in the presence of P_n_^•^ and monomers, while these propagating radicals can also be deactivated by ^•^I–catalyst, leading to the regeneration of P_n_–I–catalyst complexes. In 2021, Hou's group introduced a novel catalyst, amphiphilic ethidium iodide (EI), with broadband light absorption capabilities for photoinduced RCMP (Figure 8, #15; Table 2, #20).^[^ ^25l^ ^]^ While the previous RCMP system from 2015 demonstrated successful polymerization in bulk or using diglyme as the solvent, this new system utilizing EI as a catalyst allows for controlled radical polymerization of hydrophilic methacrylates in water under blue, green, red, and NIR light irradiation. The broadband‐absorbing nature of EI enables efficient solar energy utilization for well‐defined polymer synthesis. The system also showcased excellent temporal control, as demonstrated by the absence of monomer conversion in darkness.

**Figure 18:**
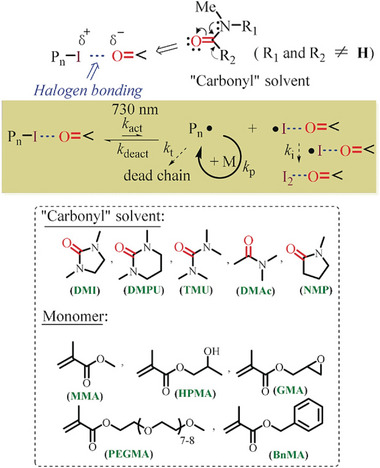
Proposed polymerization mechanism of photo‐RCMP using carbonyl solvents as catalysts under NIR light irradiation. Reproduced with permission.^[^ ^25j^ ^]^ Copyright 2020, John Wiley & Sons.

Cheng and colleagues conducted a study on a photoinduced RCMP system that does not require the addition of amines as catalysts. Instead, they used solvents containing carbonyl groups, namely, 1,3‐dimethyl‐2‐imidazolidinone (DMI), tetramethylurea (TMU), 1,3‐dimethyl‐tetrahydropyrimidin‐2(1*H*)‐one (DMPU), *N,N*‐dimethylacetamide (DMAc), and *N*‐methyl pyrrolidone (NMP), to control this RCMP (Figure 8, #16; Table 2, #21).^[^ ^25j^ ^]^ The presence of carbonyl groups in these solvents allows for the formation of interactions with the alkyl iodide, facilitating the polymerization process without the need for additional amine catalysts. The interaction between the carbonyl group of the solvents and P_n_–I leads to the formation of a halogen bond, promoting the cleavage of P_n_–I, the cleavage of P_n_–I–OR_1_R_2_ into P_n_^•^ and I–O R_1_R_2._^[^ ^79^ ^]^ This process enables a reversible equilibrium of the generation of propagating species in the photopolymerization under NIR light (**Figure** **18**).^[^ ^53^, ^80^ ^]^ Furthermore, well‐defined polymethacrylates were successfully synthesized even when nontransparent barriers were placed between the light source and reaction solution. In 2021, Cheng ’s group demonstrated that sodium iodide (NaI) can work as a catalyst,^[^ ^25^, ^81^ ^]^ in addition to “carbonyl” solvents, in RCMP. The formation of halogen bonds is more efficient in the presence of higher concentrations of NaI, promoting the cleavage of the C─I bond and resulting in an accelerated rate of RCMP. Additionally, excellent temporal control and oxygen tolerance were also demonstrated in this RCMP system. In another recent study conducted by the same research group,^[^ ^54^ ^]^ the authors identified certain electron‐deficient species, such as 1,4,5,8‐naphthalenetetracarboxylic dianhydride (NTCDA; Figure 8, #17), as highly efficient catalysts for mediating RCMP in the presence of common reducing agents, like sodium ascorbate, under NIR irradiation. According to the proposed mechanism, NTCDA is initially reduced by sodium ascorbate, leading to the generation of NTCDA anionic radical (NTCDA•−), which subsequently initiates the cleavage of the C─I bond within the complex (P_n_–I–NTCDA•−), thereby producing Pn• for mediating RCMP (Table 2, #22).

### 2.3 NIR‐RDRP via Photothermal Conversion

**Figure 19:**
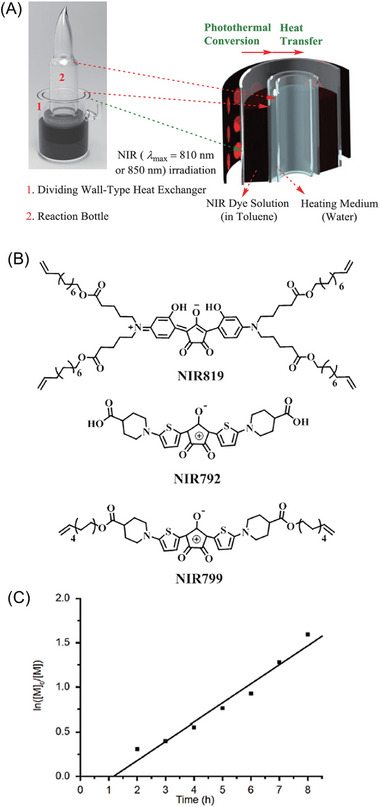
A) Scheme of dividing wall‐type heat exchanger via photothermal conversion induced by NIR‐absorbing dye solution under NIR light irradiation. B) Structures of ketocyanine‐type dyes: NIR819, NIR792, and NIR799 used in this work. C) ln([M]_0_/[M]) versus irradiation time in RAFT polymerization via photothermal conversion. Reproduced with permission.^[^ ^26^ ^]^ Copyright 2021, Springer Nature.

In 2021, Cheng's group utilized a series of ketocyanine‐type dyes that exhibit NIR absorption (**Figure** **19**B) for photothermal conversion to induce RDRP in the presence of thermal initiators. They developed a strategy that involved a heat exchanger setup (Figure 19A) consisting of NIR dye solution (for photothermal conversion), heating transfer medium, and inner reaction solution for polymerization.^[^ ^26^ ^]^ Under 100 mW cm^−2^ NIR light irradiation, the reaction temperature of the polymerization solution went up from 25 to 60 °C in 15 min, enabling the efficient homolysis of thermal initiator azobisisobutyronitrile (AIBN). This innovative NIR‐RDRP system demonstrated controlled characteristics of the RAFT polymerization, as supported by kinetic studies (Figure 19C). Importantly, this strategy proved effective mediation of other RDRP techniques, such as ATRP and RCMP under NIR irradiation. In the recycling experiments, although ≈34% of NIR dyes were photobleached after 196 h, here was no significant change in the efficiency of photothermal conversion after ten heating–cooling cycles. This finding confirms the high photostability of the dyes used in the NIR‐RDRP system, suggesting their suitability for prolonged and repetitive applications. Recently, a successful application of this photothermal system involved doping NIR‐absorbing dyes into silica nanoparticles as photosensitizers for the fabrication of thermosensitive hydrogels.^[^ ^82^ ^]^ In this process, temperature‐sensitive monomers, including an ionic liquid monomer (TVBP), *N*‐isopropylacrylamide (NIPAM), and diacrylate containing poly(propylene glycol), were successfully copolymerized along with the crosslinker for the preparation of hydrogels. Notably, the ionic liquid monomer TVBP can generate relatively high osmotic pressure, allowing the hydrogels to draw fresh water from brackish water through a semipermeable membrane. This innovative approach enables efficient recovery of fresh water from hydrogels through photothermal conversion under NIR LED irradiation.

### 2.4 NIR‐RDRP via Two‐Photon Absorption

**Figure 20:**
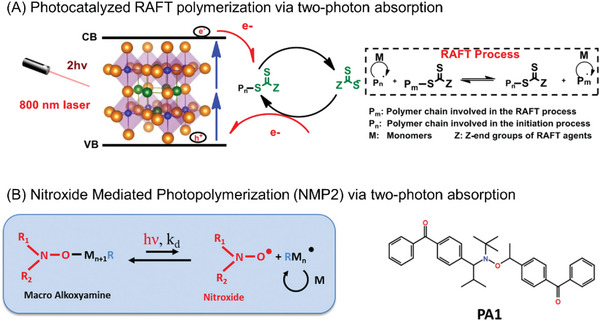
A) Proposed mechanism of photoinduced RAFT polymerization catalyzed by CsPbBr_3_ NCs via two‐photon absorption. Reproduced with permission.^[^ ^27a^ ^]^ Copyright 2020, American Chemical Society. B) Nitroxide‐mediated photopolymerization (NMP2) via two‐photon absorption. Reproduced with permission.^[^ ^18^ ^]^ Copyright 2023, John Wiley & Sons.

Egap and co‐workers introduced the first NIR‐RDRP system via TPA in 2020.^[^ ^27a^ ^]^ Unlike single‐photon absorption, this system requires the use of NIR femtosecond laser pulses.^[^ ^83^ ^]^ The researchers exploited the remarkable two‐photon absorption cross‐section of CsPbBr_3_ perovskite nanocrystals to activate PET‐RAFT polymerizations (**Figure** **20**A) under the irradiation of femtosecond laser pulses centered at 800 nm. This approach allowed for the synthesis of well‐defined poly(methyl acylate). Control experiments in the absence of perovskite nanocrystals demonstrated minimal monomer conversion, confirming that the polymerization was not initiated by the photolysis of RAFT agents. In 2023, Spangenberg and co‐workers utilized alkoxyamine PA1 (R‐O‐NR_1_R_2_) bearing benzophenone groups as both a photoinitiator and a control agent for nitroxide‐mediated photopolymerization (NMP2) via a two‐photon absorption under irradiation of NIR (720, 740, and 760 nm) femtosecond laser pulses.^[^ ^18^ ^]^ Under light excitation, R‐O‐NR_1_R_2_ undergoes homolysis of the C─O bond to generate carbon initiating radicals (R^•^) and a stable nitroxide radical (^•^O‐NR_1_R_2_).^[^ ^84^ ^]^ Chain propagation occurs in the presence of R^•^ species and vinyl monomers, resulting in the formation of polymer radicals R‐M_n_^•^. R‐M_n_^•^ species can be reversibly deactivated by ^•^O‐NR_1_R_2_ to generate macro‐alkoxyamine (R‐M_n_‐O‐NR_1_R_2_) (Figure 20B). The reversible deactivation of initiating radicals enables polymerization in a controlled manner. Like R‐O‐NR_1_R_2_, these R‐M_n_‐O‐NR_1_R_2_ macro‐alkoxyamines can fragment to generate radicals and ^•^O‐NR_1_R_2_ again under light irradiation. This NIR light mediated NMP2 system was successfully applied to 3D two‐photon laser printing (2PLP) method, which will be introduced in the following section.

## 3 Application of NIR‐RDRP

**Figure 21:**
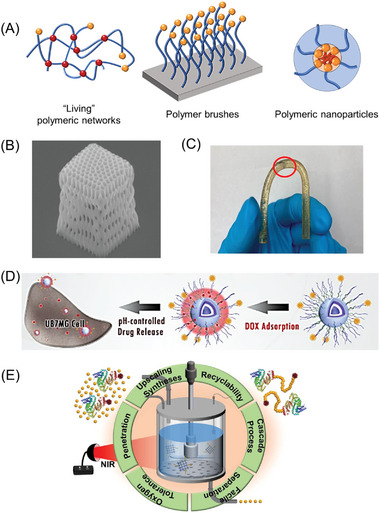
Applications of NIR‐RDRP. A) Utilization of NIR‐RDRP for the preparation of polymer materials with advanced topologies and compositions, including polymeric networks, polymer brushes, and polymeric nanoparticles. B) Fabrication of objects via 3D printing. C) Preparation of healable hydrogel preparation. D) Development of nanohybrids for drug delivery. E) Synthesis and purification of protein–polymer bioconjugates using membrane reactors.

The RDRP technique empowers the straightforward synthesis of polymers with intricate and well‐defined architectures. In contrast to other phtoRDRP activated by visible light, NIR‐RDRP processes enable the synthesis of polymer materials even when confronted with nontransparent barriers. Moreover, the longer wavelengths inherent to NIR light contribute to reduce light scattering, facilitating the practice of photo‐RDRP within colloidal reaction media. Additionally, the low energy of NIR light minimizes potential damage to sensitive systems, such as cells during photopolymerization, renders these extended wavelengths highly suitable for the preparation of bioactive materials. NIR‐RDRP represents a convergence of the unique properties of NIR light with the precision control offered by RDRP, resulting in a multitude of promising applications (**Figure** **21**).

### 3.1 NIR‐RDRP for Polymer Material Synthesis

#### 3.1.1 Polymeric Networks with “Living” Properties

Numerous NIR‐RDRP systems have found extensive application in the creation of polymeric networks. The advantage lies in the capacity to synthesize these networks through nontransparent barriers, facilitated by the enhanced light penetration afforded by longer NIR wavelengths. Furthermore, a key feature of these networks is the retention of high‐functionality groups within the polymer chains, achieved through RDRP processes such as RAFT polymerization. These thiocarbonylthio groups confer “living” properties to the synthesized polymeric networks (Figure 21A). These embedded functional groups within the networks can be reactivated for subsequent photopolymerization under NIR irradiation, opening up exciting possibilities in 3D printing and the development of healable hydrogels, as discussed in greater detail in Sections 3.2 and 3.3.

#### 3.1.2 Surface‐Initiated Polymer Brushes

**Figure 22:**
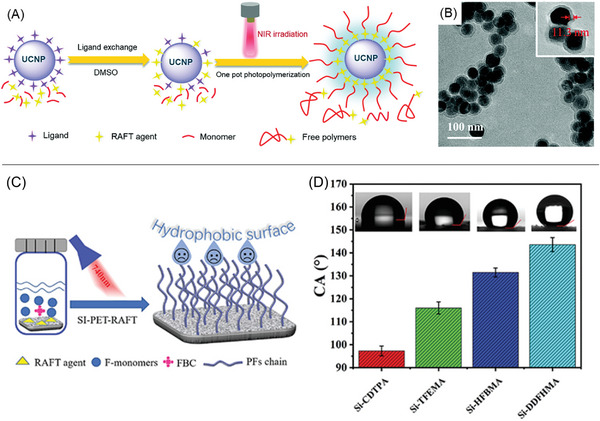
A) Scheme of the preparation of polymer brushes on the surface of UCNPs by the “grafting from” approach via NIR‐RAFT polymerization. B) TEM images of UCNP@PMMA after the polymerization for 36 h. (A,B) Reproduced with permission.^[^ ^24f^ ^]^ Copyright 2021, Royal Society of Chemistry. C) Scheme of grafting polymer brushes via SI‐PET‐RAFT polymerization under NIR light. D) The contact angle of the Si‐CDTPA, Si‐TFEMA, Si‐HFBMA, and Si‐DDFHMA. SEM and AFM images of PVA and PVA‐*g*‐PCBMA hydrogel. (C,D) Reproduced with permission.^[^ ^50^ ^]^ Copyright 2022, John Wiley & Sons.

Leveraging the enhanced penetration of NIR light, NIR‐RDRP can be utilized to fabricate polymer brushes through opaque barriers, allowing the chemical and physical properties of interfaces to be tailored. Pang's group prepared polymer brushes in situ on the surface of UCNPs via NIR‐RAFT polymerization, providing a new method for producing complex hybrid nanoparticles via photopolymerization.^[^ ^24f^ ^]^ In this approach, monodisperse β‐NaYF4:Yb/Tm UCNPs were synthesized by a solvothermal method, which was followed by surface functionalization using RAFT agents (**Figure** **22**A).^[^ ^85^ ^]^ Under the irradiation of the NIR laser, UCNP emitted blue light enabling the activation of RAFT agents and polymerization via the photoiniferter RAFT process.^[^ ^86^ ^]^ In addition, the remarkable penetration of NIR light at 980 nm has been demonstrated using biological tissue, such as chicken skin (Figure 22B).

In another study, Cao and co‐workers utilized surface‐initiated PET‐RAFT (SI‐PET‐RAFT) polymerization for the fabrication of fluorinated hydrophobic surfaces on the silicon wafer.^[^ ^50^ ^]^ The authors attached RAFT agents to the silicon wafer by reacting *N*‐hydroxysuccinimide‐capped RAFT agents (RAFT‐NHS) with Si‐NH_2_ present on the silicon wafer.^[^ ^87^ ^]^ Various polymer brushes, including poly(trifluoromethyl methacrylate), poly(hexafluorobutyl methacrylate), poly(dodecafluoroheptyl methacrylate), were successfully prepared on a silicon wafer under NIR light irradiation (Figure 22C). The surface angle varied from 99.5° to 118°–146.5° after grafting different types of polymer brushes on silicon (Figure 22D), indicating the facile adjustment on the hydrophobicity of silicon substrate via SI‐PET‐RAFT polymerization.

**Figure 23:**
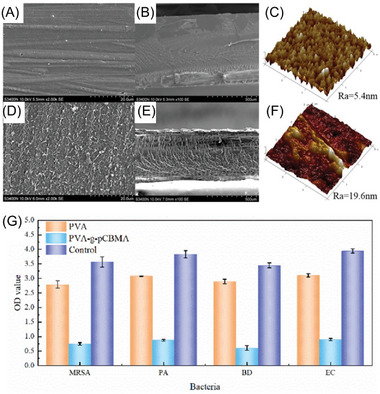
A) SEM image of PVA. B) the cross‐section SEM image of PVA. C) AFM image of PVA. D) SEM image of PVA‐*g*‐PCBMA. E) the cross‐section SEM image of PVA‐*g*‐PCBMA. F) AFM image of PVA‐*g*‐PCBMA. G) Antifouling properties of hydrogel: bacterial adhesion. Note: *Methicillin‐resistant Staphylococcus aureus* (MRSA), *Pseudomonas aeruginosa* (PA), *Acinetobacter baumannii* (AB), and *Escherichia coli* (EC) were selected. Reproduced with permission.^[^ ^33c^ ^]^ Copyright 2021, Elsevier.

In 2021, Hu and co‐workers employed NIR light‐induced RAFT polymerization for the attachment of CBMA polymer brushes on the PVA hydrogels, enhancing the antifouling performance.^[^ ^33c^ ^]^ A smooth surface of PVA (**Figure** **23**A,B) was successfully modified to form wrinkle morphology after grafting PCBMA (Figure 23D,E) as indicated by SEM images. The PVA surface appeared needle‐like with a root‐mean‐square surface roughness (*R*_a_) of ≈5.4 nm (Figure 23C), while the *R*_a_ value rose to ≈19.6 nm after the attachment of CBMA polymer (Figure 23F), indicating a noticeable transformation in surface topography indicating the successful functionalization of the hydrogel surface. The grafting of PCBMA onto PVA hydrogel increases the surface hydration effect via electrostatic interaction and enhances the steric repulsion effect, hindering the protein adsorption on the surface of PVA‐*g*‐PCBMA.^[^ ^88^ ^]^ This leads to a significant improvement in the prevention of bacteria adhesion (Figure 23G).

#### 3.1.3 Polymeric Nanoparticles

**Figure 24:**
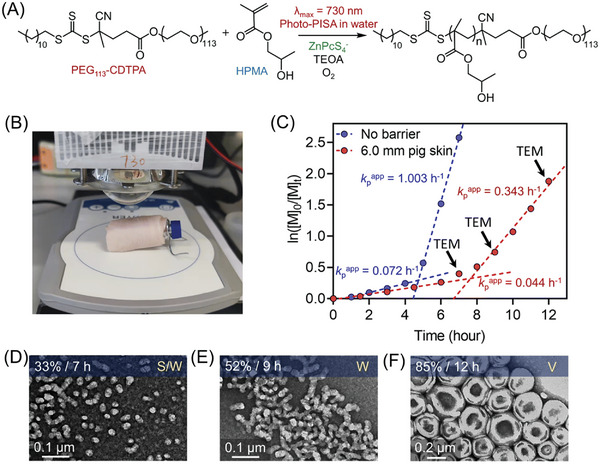
A) Scheme and B) experimental setup of photo‐RAFT dispersion polymerization under the NIR light (*λ*_max_ = 730 nm; 60 mW cm^−2^) irradiation passing through 6.0 mm pig skin. C) Comparison of dispersion photo polymerization kinetics between without barrier and through 6.0 mm pig skin. D–F) Evolution of morphologies of polymeric nanoparticles synthesized through 6.0 mm pig skin indicated by corresponding TEM images at different time points (*t* = 7, 9, and 12 h). Reproduced with permission.^[^ ^17c^ ^]^ Copyright 2022, Royal Society of Chemistry.

In dispersion photopolymerization, incident light with longer wavelengths (higher *λ*) can be considered an ideal light source, especially for the synthesis of nanoparticles with large diameters (*d*). As light scattering is proportional to *d* ^6^/*λ* ^4^, the scattering decreases with longer‐wavelength incident light, enabling a more even distribution of light intensity in colloidal media. This can benefit the upscaling of the production of polymeric nanoparticles via dispersion photopolymerization. Recently, NIR‐RDRP systems have been successfully applied in photoinitiated polymerization‐induced self‐assembly (photo‐PISA) systems.^[^ ^89^ ^]^ For instance, our group reported an aqueous NIR‐RAFT system for the first application in the synthesis of polymeric nanoparticles via photo‐PISA.^[^ ^17c^ ^]^ In the presence of TEOA and ZnPcS_4_^−^ under NIR irradiation, dissolved oxygen was transformed into hydrogen peroxide efficiently, enabling successful photo‐RAFT polymerization without deoxygenation. Photo‐RAFT mediated aqueous dispersion polymerization of 2‐hydroxypropyl methacrylate was successfully conducted under NIR irradiation using a poly(ethylene glycol) (PEG)‐functionalized RAFT agent (PEG_113_‐CDTPA) as the first stabilizing block (**Figure** **24**A). By varying the targeted degree of polymerization, nanoparticles with diverse morphologies, including spheres, worms, and vesicles, were successfully prepared. Exploiting the high light penetration of longer wavelengths, photo‐PISA was successfully induced when an opaque barrier was introduced (Figure 24B,C). In this process, the evolution of nanoparticle morphologies, from spheres to vesicles, was observed with increasing light exposure time (Figure 24D–F) and was not affected by the presence of an opaque barrier. More specifically, at the final point of kinetics, consistent vesicle morphologies with a diameter of ≈200 nm of polymeric nanoparticles were successfully prepared by photo‐PISA through a 6.0 mm thick pig skin barrier (Figure 24F).

In another example, Cao and co‐workers developed a PET‐RAFT polymerization system catalyzed by fluorophenyl bacteriochlorin under NIR irradiation.^[^ ^50^ ^]^ This system was also applied in the synthesis of polymeric nanoparticles using the photo‐PISA process. Experimentally, poly(oligo(ethylene glycol) methyl ether methacrylate) macro‐RAFT agent was first synthesized as the hydrophilic stabilizing block for subsequent photo‐PISA reactions in ethanol. Dispersion PET‐RAFT polymerization of two semifluorinated monomers was conducted under NIR irradiation. With increasing monomer conversion in the photo‐PISA process, the transparent reaction mixture turned into a colloidal solution with a milky appearance. The successful formation of nanoparticles was demonstrated by DLS and TEM characterization.

### 3.2 3D Printing and Postmodification of Printed Objects

The utilization of photo‐RDRP techniques for 3D printing allows the synthesis of polymers with high retention of functionalized groups (such as thiocarbonylthio or alkoxyamine groups) embedded within printed polymeric objects. These groups can be easily reactivated or modified after printing, enabling further alteration of the properties of 3D printed materials.^[^ ^9b‐d^ ^]^ Compared to UV and visible light, NIR wavelengths possess enhanced penetration capabilities, facilitating deep photocuring in the 3D printing process.

**Figure 25:**
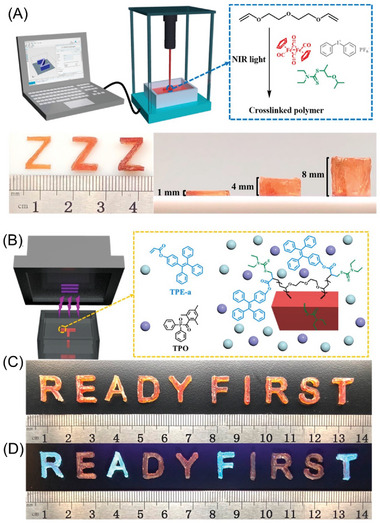
A) 3D printing regulated by NIR light via a photoinduced free radical‐promoted cationic RAFT polymerization and printed objects with different thicknesses. B) Surface functionalization of the 3D printed objects C) under natural light and D) under UV light. Reproduced with permission.^[^ ^20^ ^]^ Copyright 2021, American Chemical Society.

In 2021, Zhu and co‐workers developed an NIR light‐regulated photoinduced cationic RAFT polymerization regulated using FeCp(CO)_2_Br as the photosensitizer and diphenyliodonium salt as the initiator (Figure 14B).^[^ ^20^ ^]^ This system exhibited excellent tolerance toward oxygen,^[^ ^90^ ^]^ allowing for successful application in 3D printing under open‐air conditions using a 788 nm NIR laser diode (**Figure** **25**A). Leveraging the enhanced penetration of NIR light, objects of varying thicknesses could be printed. To demonstrate this, single‐layer printed objects in the shape of the letter “Z” were created with heights of 1, 4, and 8 mm (Figure 25A). Due to the high retention of thiocarbonylthio groups at the end of polymer chains, the printed objects can be postfunctionalized via chain extension. Using this NIR approach, two words, namely, “READY” and “FIRST,” were successfully 3D printed. Subsequently, “R”, “A”, “F”, and “T” of “READY FIRST” were functionalized using a fluorescent monomer, tetraphenylethylene‐acrylate (TPE‐a) via photo‐RAFT polymerization in the presence of photoinitiator phosphine oxide under UV irradiation (Figure 25B). Consequently, all letters exhibited a uniform color when exposed to natural light (Figure 25C), but under UV light, only the letters “RAFT” emitted a fluorescent blue light (Figure 25D), demonstrating the successful chain extension (Figure 25D). The successful surface functionalization via this method indicates its potential application in the preparation of anticounterfeit materials. By contrast, in the absence of RAFT agents, no fluorescence was observed after postmodification.

**Figure 26:**
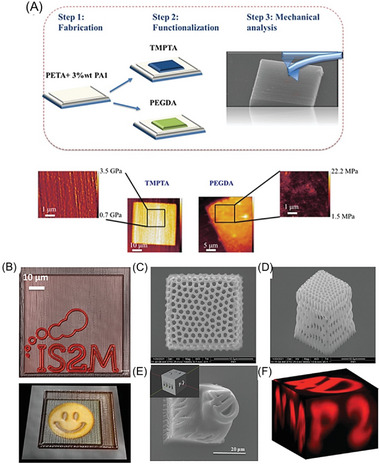
A) Postmodification and characterization of modified materials: mechanical properties changed over 2 orders of magnitude regarding the chosen monomer for functionalization (TMPTA or PEGDA). B) Customization and successive surface modification of printed microstructures made by 3D two‐photon laser printing via NMP2 and visualized by fluorescence and reflection confocal microscopies. C) Top and D) tilted SEM images of Voronoi 3D microstructures. E) Fabrication of a cubic structure and its postmodification by “4D”, “NM”, and “P2” letters on its surface shown in CAD and SEM images. F) 3D fluorescence reconstruction imaging of the functionalized cubic structures with fluorescent TMPTA‐based formulation. Reproduced with permission.^[^ ^18^ ^]^ Copyright 2023, John Wiley & Sons.

Recently, Spangenberg and co‐workers reported a 3D two‐photon laser printing (2PLP) using NMP2.^[^ ^18^ ^]^ It is worth mentioning that they employed an NIR femtosecond pulsed laser as the light source for achieving 3D printing via two‐photon absorption. Unlike the one‐photon system previously reported by Zhu's group, which employed continuous wave lasers, 2PLP allows for the precise fabrication of high‐resolution objects, typically on a micrometer scale. In this process, an alkoxyamine compound containing two benzophenone groups (PA1) served both as a photoinitiator and a control agent within a solvent‐free photoresist. This innovative approach enabled 3D printing via NMP2 using the 2PLP technique (Figure 20B). The presence of alkoxyamine groups within the polymeric networks allows for the postfunctionalization of printed objects through chain extension. In an experimental setup, a polymer square composed of pentaerythritol triacrylate (PETA) was initially fabricated using 3D two‐photon laser printing (2PLP). Subsequently, chain extension was performed by printing a square on top of the PETA polymer square using either trimethylolpropane triacrylate (TMPTA) or poly(ethylene glycol)diacrylate (PEGDA) without the addition of any photoinitiator. This process resulted in significantly distinct topography and mechanical properties between the original PETA square and the newly printed square (**Figure** **26**A).

The living characteristics of printed objects via NMP2 enable easy modification of the mechanical properties of material surfaces as well as the surface of these objects. For instance, the storage modulus of 3D printed materials was modified over two orders of magnitude from MPa to GPa via post‐treatment. Furthermore, the high resolution attainable by this technique allowed the functionalization of the 3D printed surface (Figure 26B, top). Furthermore, this process allows for the fabrication of arbitrary microstructures. In this study, a Voronoi structure with dimensions of 20 µm × 20 µm × 20 µm was successfully constructed via NMP2‐mediated 3D printing, achieving sub‐micrometer resolution (Figure 26C,D). Excitingly, the 2PLP system via NMP2 also enabled 4D printing,^[^ ^91^ ^]^ which refers to the production of programmable objects that can transform under a specific stimulus over time after being printed (Figure 26E,F).

**Figure 27:**
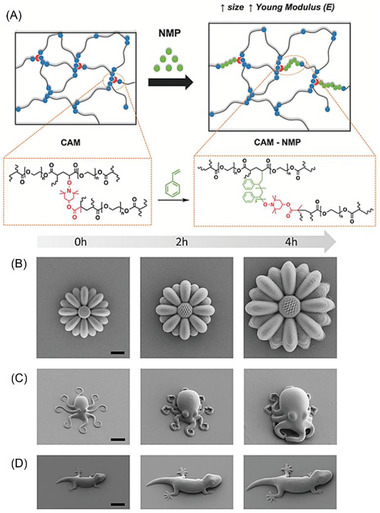
A) Alkoxyamine‐containing covalent adaptable microstructures (CAMs) prepared via two‐photon laser printing, and their postmodification via nitroxide‐mediated polymerization (CAM‐NMP). NMP chain extension of complex CAM microstructures, including B) sunflower, C) octopus, and D) gecko, with styrene at different reaction times. Scale = 20 µm for all images. Reproduced with permission.^[^ ^27b^ ^]^ Copyright 2022, John Wiley & Sons.

In another work, Blasco and co‐workers incorporated alkoxyamine compounds (TEMPO) into a conventional ink system, enabling the successful fabrication of 3D microstructures with covalent dynamic bonds.^[^ ^27b^ ^]^ The ink was composed of PEGDA, TEMPO‐methacrylate containing a stable nitroxide radical, and 7‐diethylamino‐3‐thenoylcoumarin photoinitiator, which can be printed sub‐µm resolution via 2PLP under the irradiation of NIR femtosecond pulsed laser. Although the photopolymerization process in 3D printing did not follow the RDRP process due to the irreversible deactivation between propagating radicals and nitroxide radicals at ambient temperature, printed objects are regarded as covalent adaptable microstructures (CAMs) possessing living characteristics owing to the retention of alkoxyamine. For example, 3D printed CAMs were postmodified by activating the nitroxide group at 130 °C in the presence of styrene^[^ ^92^ ^]^ under N_2_ atmospheres (**Figure** **27**A). Under this condition, alkoxyamine groups embedded in printed polymeric chains were reversibly activated to generate initiating radicals, leading to further polymerization of styrene. SEM confirmed the successful growth of the polymer network with the maintenance of the macrostructure and shape (Figure 27B–D).

### 3.3 Fabrication of Healable Hydrogels

The enhanced penetration of NIR light through opaque and nontransparent barriers presents a unique opportunity for the preparation of hydrogels via photopolymerization in a situation where visible light cannot be easily applied.^[^ ^9^ ^]^ In addition, the use of RDRP imparts “living” properties to synthesized polymer materials, enabling the reactivation of polymers via chain extension and the reshuffling of polymer chains. This combination provides the opportunity to produce healable hydrogels, which can be repaired through thick barriers. This development holds great promise for various applications in the field of hydrogel materials.

**Figure 28:**
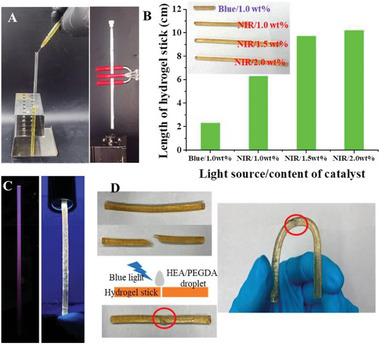
A) Experimental setup of Injection of ATRP solution to the glass tube (left) and reaction irradiated by NIR laser (right). B) Length of hydrogel sticks versus different light sources and content of catalysis. C) Dual mode photoluminescence under different irradiation light sources of NIR (left) and UV (right). D) Reparation process of hydrogel stick (left) under blue light and the performance after repair (right). Reproduced with permission.^[^ ^17b^ ^]^ Copyright 2022, American Chemical Society.

Pang and co‐workers developed composite nanoparticles, named UCNP@SiO_2_@N‐CDs, by combining N‐CDs and UCNPs, as efficient heterogeneous PCs to mediate photo‐ATRP. This system can be regulated by the broadband light range from UV/vis light to NIR (Figure 5C).^[^ ^17b^ ^]^ Moreover, great performance in aqueous media was observed, enabling the preparation of hydrogel. Experimentally, the reaction solution after deoxygenation was transferred to the glass tubes, followed by laser irradiation (**Figure** **28**A). Although successful gelation was observed both under the irradiation of blue (465 nm) and NIR (980 nm) lasers, a much longer hydrogel stick was obtained under NIR irradiation, which is attributed to higher light penetration of NIR than UV light (Figure 28B). Meanwhile, higher concentrations of PC in the system showed more efficient polymerization with longer hydrogel sticks. Moreover, the synthesized hydrogel can emit violet light under NIR irradiation and yellow light under UV irradiation (Figure 28C), which is attributed to the special photoluminescence behavior of UCNP@SiO_2_@N‐CDs. As hydrogel samples were synthesized via photo‐ATRP exhibiting a “living” character, the functional group embedded in polymeric networks could be reactivated. In the hydrogel reparation experiment, a small amount of 2‐hydroxyethyl acrylate/poly(ethylene glycol) diacrylate solution was added to the incision, which was followed by blue light irradiation. Chain extension was successfully performed with fresh monomers, enabling the reformation of hydrogel sticks (Figure 28D).

**Figure 29:**
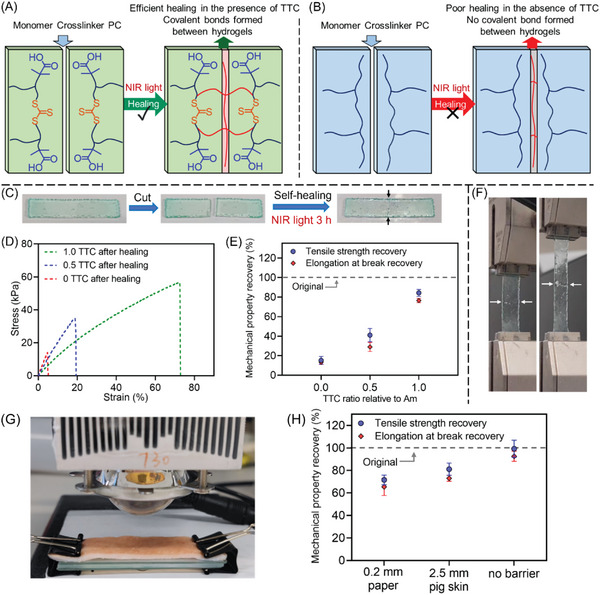
A) The healing process of hydrogels containing RAFT agents by photo‐RAFT polymerization under NIR light. B) Limited healing of FRP‐mediated hydrogels under NIR light. C) Digital photos of the hydrogel healing experiment. D) Stress–strain curves of healed hydrogels containing various concentrations of TTC RAFT agent under NIR light for 3 h. E) Tensile property recovery of healed hydrogels containing various concentrations of TTC RAFT agent (0, 0.5, and 1 ratios relative to 300 units of Am) under NIR light for 3 h. F) Experimental setup of tensile testing of the healed hydrogel. G) Experimental setup of hydrogel healing in a mold consisting of a silicone frame and two glass plates under NIR light passing through 2.5 mm pig skin. H) Mechanical property recovery of hydrogel samples after the photoinduced healing through various nontransparent barriers (0.2 mm paper and 2.5 mm pig skin) under NIR irradiation for 5 h. Reproduced with permission.^[^ ^17a^ ^]^ Copyright 2023, John Wiley & Sons.

However, this NIR‐ATRP system developed by Pang's group required prior deoxygenation for photopolymerization and preparation of hydrogels. Furthermore, the hydrogel samples were only healed under blue light. These limitations were recently overcome by our group. We reported an aqueous NIR‐RAFT system catalyzed by tetrasulfonated zinc phthalocyanine (ZnPcS_4_^−^) in the presence of peroxides for the preparation of hydrogels^[^ ^17a^ ^]^ (**Figure** **29**A), exhibiting excellent oxygen tolerance.^[^ ^17a^ ^]^ Without the need for prior deoxygenation, photoinduced gelation was efficiently performed through nontransparent barriers. The “living” character of synthesized hydrogels enabled the on‐demand reactivation of polymer end groups under NIR irradiation (Figure 29A), which was demonstrated by a healing experiment. Before placing broken samples under NIR light for 3 h, 20 µL of hydrogel precursor solution without RAFT agent was added to the interface (as illustrated in Figure 29C). The tensile testing results of the healed hydrogel samples exhibited a significant recovery in their mechanical properties. The healed hydrogels exhibited an 84 ± 4% recovery of the tensile strength and 76 ± 2% of the elongation at break (Figure 29D–F). These improvements can be attributed to the RAFT chain transfer process and crosslinking copolymerizations that take place during the healing process. On the contrary, hydrogels prepared by free radical polymerization (FRP) only displayed minimal healing ability in the presence of the same precursor solution, performing less than 16 ± 4% and 13 ± 2% recovery of stress and elongation at break. The poor healing performance of FRP‐mediated hydrogels was attributed to the absence of reactivation of functional groups in these networks and no covalent bonds formed across isolated hydrogel samples (Figure 29B). Owing to the enhanced light penetration of NIR light, the photoinduced healing process demonstrated a high recovery of hydrogel tensile strength even when healing through various nontransparent barriers (Figure 29G,H).

### 3.4 Preparation of Nanohybrids for Drug Delivery

**Figure 30:**
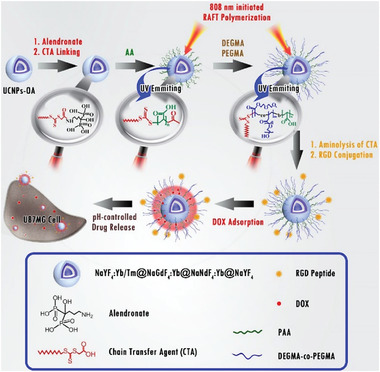
Schematic illustration of the synthesis procedure of hierarchical block copolymer brushes on UCNPs via NIR light‐induced grafting‐from RAFT polymerization and the application for drug delivery. Reproduced with permission.^[^ ^21^ ^]^ Copyright 2017, American Chemical Society.

Luan and colleagues reported an NIR‐RAFT polymerization for the construction of hierarchical polymer brushes on UCNPs.^[^ ^21^ ^]^ The synthesis procedure is presented in **Figure** **30**A. The synthesized UCNPs were performed with the ligand exchange process using alendronate, enhancing the hydrophilicity of these UCNPs. Then, the NH_2_ group at the surface of UCNPs enables anchoring RAFT agents by amide conjugation. Under the irradiation of 808 nm laser light, UCNPs emitted visible light, promoting the cleavage of thiocarbonylthio groups to activate photoiniferter RAFT polymerization. By this method, diblock copolymer brushes consisting of poly(acrylic acid) (PAA) and poly(oligo(ethylene oxide)methacrylate‐*co*‐2‐(2‐methoxy‐ethoxy)ethyl methacrylate) were successfully grafted on the surface of UCNPs. Subsequently, UCNPs were modified with arginine–glycine–aspartic (RGD) peptide, which provides targeting capability to cancer cells. To demonstrate this capability, the UCNPs@PAA‐*b*‐PEG‐RGD group was characterized with significantly stronger luminescence signals than the UCNPs@PAA‐*b*‐PEG‐PEG_600_ control group at each given time point. These findings suggest that RGD peptide modification facilitated the endocytosis of nanoparticles.^[^ ^93^ ^]^ Finally, these hybrid nanoparticles were loaded with doxorubicin (DOX), exhibiting pH‐sensitive drug release behavior. The pH‐responsive release can be attributed to the protonation of PAA carboxyl groups in an acidic environment, reducing the electrostatic interaction and accelerating the release of DOX.^[^ ^94^ ^]^ This promotes the drug release in mildly acidic tumor tissues as well as more acidic endosomes and lysosomes after endocytosis. Moreover, the modification of RGD enhances cellular uptake in U87MG cell therapy.^[^ ^95^ ^]^ Compared to free DOX, prepared DOX‐UCNPs@PAA‐*b*‐PEG‐RGD nanohybrids improve therapeutic effect for the treatment of U87MG cancer cells, owing to the key role of RGD‐mediated endocytosis. The uptake of nanodrugs by cells can take a completely different pathway, resulting in the enhanced distribution of DOX in the cytoplasm and overcoming the limitations of conventional free drug delivery methods.^[^ ^96^ ^]^ These results display potential applications of this NIR‐RAFT system in biomedical fields owing to the low phototoxicity and deep penetration of NIR light.

### 3.5 Synthesis and Purification of Protein–Polymer Bioconjugates in Membrane Reactors

**Figure 31:**
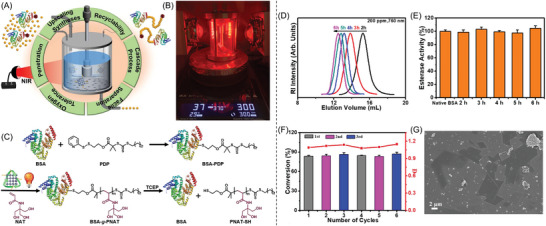
A) Schematic illustration of membrane reactors with suspended polyphthalocyanine nanoplatelets under NIR light for upscaling syntheses of protein–polymer bioconjugates. B) Experimental setup of aqueous PET‐RAFT polymerization. C) Protein–polymer bioconjugation in SCBMR. D) GPC profiles at different time points with 200 ppm catalyst under NIR light irradiation. E) Esterase activity of BSA and BSA‐*g*‐PNAT bioconjugates with different light exposure time points (normalized against native BSA). Cascade polymerization process in SCBMR:PET‐RAFT polymerization was performed in PBS by using PPc‐MPEG as catalysts without prior deoxygenation under NIR light. F) Monomer conversion and dispersity values in PET‐RAFT polymerization catalyzed by PPc‐MPEG after different numbers of reusing cycles. G) SEM image of PPc‐MPEG after 6 cycles of polymerization. Reproduced with permission.^[^ ^19^ ^]^ Copyright 2023, Elsevier.

The combination of photo‐RDRP and membrane separation techniques facilitates the production and purification of a wide variety of well‐defined macromolecules with high purity (**Figure** **31**A,B).^[^ ^97^ ^]^ In this integrated system, as NIR light involves lower energy and is less likely to cause damage in sensitive systems containing proteins or cells during photopolymerization, these long wavelengths are particularly suitable to be utilized for the preparation of bioactive materials. Cai and co‐workers synthesized a novel heterogeneous PC, named MPEG‐linked PPc‐MPEG, for PET‐RAFT polymerization under NIR light in water.^[^ ^19^ ^]^ In this study, bovine serum albumin (BSA) was employed as a model protein, which was conjugated with RAFT agents for further PET‐RAFT polymerization. A disulfide exchange reaction by reacting 2‐(pyridine‐2‐yldisulfanyl)ethyl 2‐(((dodecylthio)carbonthioyl)thio) propanoate (PDP) with a free sulfhydryl cysteine residue (Cys‐34) in BSA was carried out in a mixture of phosphate buffer solution (PBS, pH 7.4, 100 mm) and DMSO (v/v, 20:1) to obtain BSA‐PDP macrochain transfer agent (macro‐CTA) (Figure 31C). *N*‐[tris(hydroxymethyl)methyl] acrylamide (NAT) was successfully polymerized in a controlled manner via this PET‐RAFT polymerization using BSA‐PDP macro‐CTA, enabling the synthesis of well‐defined polymers with narrow MWDs (Figure 31D). Under the PET‐RAFT polymerization condition, the high bioactivity of BSA was maintained (Figure 31E). Subsequently, suspended‐catalysts‐based membrane reactors (SCBMR) were used for facile preparation and purification of protein–polymer bioconjugates, enabling the synthesis on a large scale with consistency and high control. Another advantage of this system is that suspended PCs can be easily separated and repeatedly used for successive PET‐RAFT polymerizations in the SCBMR system. The recyclability of the catalyst system was demonstrated by successive photopolymerizations (Figure 31F), while the structural stability of these heterogeneous PCs was confirmed by SEM (Figure 31G).

## 4 Summary and Outlook

In conclusion, this review classifies and summarizes the mechanisms and applications of various NIR‐RDRP systems, providing readers with a comprehensive understanding of this emerging research area. The review also highlights the advantages, challenges, and future perspectives of NIR‐RDRP in multiple applications. We hope that this review will offer valuable insights into the unique capabilities of NIR‐RDRP and serve as a guide for the development of efficient and versatile photoinitiation systems. However, one of the main limitations of NIR‐RDRP is their slow polymerization rates due to the low photon energy of NIR light, which hampers its practical implementation. To overcome this limitation, more efficient NIR‐RDRP systems using new PCs or UCNPs need to be developed. Regarding PCs, with the development of quantum chemistry in recent years, computational calculations create new opportunities for the design of PCs based on knowledge of structure–property–performance relationships.^[^ ^98^ ^]^ For example, the design of PCs with longer lifetimes and stronger redox potentials in their excited states is crucial for more efficient photoinduced electron/energy transfer, resulting in higher polymerization rates in specific NIR‐RDRP systems.^[^ ^99^ ^]^ Concerning UCNPs, those with high yields of luminescence are needed for more efficient activation of polymerization, as they can emit UV or visible light with higher intensity under identical irradiation.^[^ ^24^, ^39^ ^]^ In addition, the optimization of the photoinitiation system can allow efficient optical transmission and absorption by the photoinitiator. For example, Yagci and colleagues prepared a novel NIR photosensitizer to enhance the polymerization rates by incorporating the functional group of photoinitiator on the surface of UCNPs.^[^ ^24e^ ^]^

As the prior deoxygenation or utilization of glove boxes complicates the production of polymers, the development of oxygen‐tolerant NIR‐RDRP is significant for their applications in broader fields. Compared to UCNP‐mediated systems, more oxygen‐tolerant systems have been demonstrated in PC‐mediated polymerization due to their ability to convert oxygen into ROS, such as singlet oxygen (^1^O_2_), superoxide, and hydrogen peroxide.^[^ ^17^, ^47^ ^]^ These ROS can be subsequently consumed, resulting in oxygen‐tolerant polymerization. Consequently, well‐defined polymers were successfully prepared under NIR irradiation without the need for prior deoxygenation. However, the oxygen conversion to ROS is relatively slow using NIR PCs in comparison to visible light or UV PCs. Therefore, there is a need to optimize the physical properties of PCs, which can be tailored by the introduction of functional groups. For example, the singlet oxygen quantum yield of PC can be increased by the incorporation of heavy atoms into a photosensitizer, resulting in a more efficient transformation from O_2_ to ^1^O_2_.^[^ ^100^ ^]^

In NIR‐RDRP, it is challenging and complex to recycle homogenous PC after completing photopolymerization. Sometimes, these PCs are attached to synthesized polymers, leading to color change and impurity involvement in the final product. Therefore, the development of heterogenous PCs with high performance and stability is promising. These suspended PCs can be facilely recovered simply by centrifuge. Importantly, heterogeneous PCs have been successfully combined with the membrane separation technique in NIR‐RDRP for the convenient production and purification of bioactive materials.^[^ ^19^ ^]^ In this process, the suspended PCs maintained good stability and integrity during the polymerization after reuse in membrane reactors multiple times. There is a need for the development of processes that can efficiently recycle PCs.

Besides fundamental studies, perspectives on applications of NIR‐RDRP are briefly discussed here. As in colloidal solutions, light scattering leads to a high gradient of light intensity through the reactor, a promising application of NIR‐RDRP is their implementation in dispersion photopolymerization for the preparation of functional polymers and nanoparticles.^[^ ^17^, ^19^ ^]^ The photo‐PISA is one representative system, which enables the preparation of polymeric nanoparticles under light irradiation. In this process, polymeric nanoparticles are generated, and their sizes increase during photopolymerization, resulting in high light scattering through reaction media. Another colloidal system is suspended‐PCs‐catalyzed photo‐RDRP where dispersed PC nanoparticles can hinder light penetration through reaction solution. Compared to UV and visible light, lower light scattering afforded by NIR wavelengths results in a more even distribution of light intensity in colloidal media. Therefore, NIR‐RDRP systems are particularly significant for upscaling the production of functional polymers in colloidal media.

3D two‐photon laser printing (2PLP) and photocuring benefit from the NIR‐RDRP systems.^[^ ^18^, ^20^, ^27^, ^33^ ^]^ On the one hand, utilization of long wavelengths promotes light penetration through the photocurable resin, facilitating the fabrication of complex microstructure with relatively high thickness in 2PLP as well as the photocuring with high depths.^[^ ^20^ ^]^ On the other hand, RDRP imparts the “living” properties to printed or cured objects, enabling facile postmodification via the reactivation of functional groups embedded in polymeric networks. For example, Spangenberg and co‐workers utilized the RDRP technique in 2PLP for the modification of printed material surfaces over two orders of magnitude from MPa to GPa.^[^ ^18^ ^]^ These advanced materials with “living” characteristics and high resolution (normally µm scale) are promising to be applied in various high‐tech fields, including microelectronics, microrobotics, and biomedicine. However, some challenges exist in the application of NIR‐RDRP systems in 3D printing techniques. First, current NIR‐RDRP systems mainly absorb relatively shorter wavelengths of NIR light (≈750 nm) for 3D printing. The penetration of these wavelengths is relatively limited compared to the wavelengths above 850 nm, leading to the deformation of printed objects due to light penetration through a large object volume.^[^ ^18^ ^]^ To solve this issue, the design of novel and efficient photosensitizers with longer‐wavelength absorption NIR‐RDRP systems with high quantum yield becomes a promising strategy. Moreover, as only a photoinduced NMP system was successfully applied in 2PLP, other photo‐RDRP systems, such as photo‐ATRP and photo‐RAFT polymerization, may have good performance for the fabrication of microstructures via 2PLP, which require further investigation by researchers.

Transdermal photopolymerization was first introduced in 1999,^[^ ^12^ ^]^ poly(ethylene glycol) dimethacrylate water solution was utilized as the precursor of hydrogel for minimally invasive implantation. Hydrogels were successfully fabricated through biological barriers in the presence of a photoinitiator under visible light. Recently, Chen et al. developed a UCNP coated with UV/blue‐light photoinitiator lithium phenyl‐2,4,6‐trimethylbenzoylphosphinate for noninvasive in vivo 3D bioprinting under NIR light.^[^ ^101^ ^]^ Exploiting high penetration of NIR light, customized tissue constructs can be formed through the noninvasive printing of subcutaneously injected bioink. However, these transdermal photopolymerization systems are mediated by free radical polymerization, limiting the control and functionalization of these polymeric networks. By contrast, NIR‐RDRP systems enable the synthesis of well‐defined polymers with complex architectures, which can be employed in the preparation of networks holding potential for applications in transdermal photopolymerization. We can expect that a broader range of advanced materials will be fabricated via NIR‐RDRP in the future development of transdermal photopolymerization. Although many current studies have demonstrated successful controlled radical polymerization when nontransparent barriers were placed between the reaction vessel and NIR light source, NIR‐RDRP has not been applied for transdermal photopolymerization in vivo. To promote the application of NIR‐RDRP in injectable hydrogel for noninvasive implantation and bioprinting, aqueous and biocompatible systems with fast polymerization rates need to be developed in the future.

## Conflict of Interest

The authors declare no conflict of interest.
